# Upper Girdle Imaging in Facioscapulohumeral Muscular Dystrophy

**DOI:** 10.1371/journal.pone.0100292

**Published:** 2014-06-16

**Authors:** Giorgio Tasca, Mauro Monforte, Elisabetta Iannaccone, Francesco Laschena, Pierfrancesco Ottaviani, Emanuele Leoncini, Stefania Boccia, Giuliana Galluzzi, Marco Pelliccioni, Marcella Masciullo, Roberto Frusciante, Eugenio Mercuri, Enzo Ricci

**Affiliations:** 1 Don Carlo Gnocchi Onlus Foundation, Milan, Italy; 2 Institute of Neurology, Catholic University School of Medicine, Rome, Italy; 3 Department of Radiology, Istituto Dermopatico dell'Immacolata, IRCCS, Rome, Italy; 4 Institute of Hygiene-Department of Public Health, Catholic University School of Medicine, Rome, Italy; 5 Cellular Biology and Neurobiology Institute, CNR, Rome, Italy; 6 Unione Italiana Lotta Distrofia Muscolare (UILDM) Rome Section, Rome, Italy; 7 IRCCS Santa Lucia Foundation, Rome, Italy; 8 Neurophysiopathology Unit, Columbus Hospital, Catholic University School of Medicine, Rome, Italy; 9 Pediatric Neurology Unit, Catholic University School of Medicine, Rome, Italy; IRCCS-Policlinico San Donato, Italy

## Abstract

**Background:**

In Facioscapulohumeral muscular dystrophy (FSHD), the upper girdle is early involved and often difficult to assess only relying on physical examination. Our aim was to evaluate the pattern and degree of involvement of upper girdle muscles in FSHD compared with other muscle diseases with scapular girdle impairment.

**Methods:**

We propose an MRI protocol evaluating neck and upper girdle muscles. One hundred-eight consecutive symptomatic FSHD patients and 45 patients affected by muscular dystrophies and myopathies with prominent upper girdle involvement underwent this protocol. Acquired scans were retrospectively analyzed.

**Results:**

The trapezius (100% of the patients) and serratus anterior (85% of the patients) were the most and earliest affected muscles in FSHD, followed by the latissimus dorsi and pectoralis major, whilst spinati and subscapularis (involved in less than 4% of the patients) were consistently spared even in late disease stages. Asymmetry and hyperintensities on short-tau inversion recovery (STIR) sequences were common features, and STIR hyperintensities could also be found in muscles not showing signs of fatty replacement. The overall involvement appears to be disease-specific in FSHD as it significantly differed from that encountered in the other myopathies.

**Conclusions:**

The detailed knowledge of single muscle involvement provides useful information for correctly evaluating patients' motor function and to set a baseline for natural history studies. Upper girdle imaging can also be used as an additional tool helpful in supporting the diagnosis of FSHD in unclear situations, and may contribute with hints on the currently largely unknown molecular pathogenesis of this disease.

## Introduction

Muscle MRI has lately been increasingly used in clinical and research practice because of its ability to detect the involvement of individual muscles including those that cannot be easily explored on clinical grounds [1]. This has led to the description of several patterns of involvement, i.e. combinations of affected and spared muscles, which in some cases are specific for certain disorders [2]. So far the vast majority of the studies have focused on the involvement of pelvic and lower limb muscles [3], likely because most muscle diseases affect more prominently the pelvic rather than the scapular girdle. Thus, whilst standardized protocols for lower limbs have been proposed and the possibility of comparison with previously described patterns exists, less is available for the assessment of upper girdle in myopathies with predominant involvement of scapular and arm muscles such as Facioscapulohumeral muscular dystrophy (FSHD) [4].

FSHD is an autosomal dominant disease whose genetic background has been clarified as a permissive condition that allows the stable transcription of the *DUX4* retrogene, which is normally silenced in adult skeletal muscle [5]. Nonetheless, some evidences suggest that this background is necessary but not sufficient *per se* to cause the disease since the permissive haplotype can be found in the healthy population with a relatively high frequency compared to the incidence of FSHD [6]. Thus, the diagnosis of FSHD has to be made by a combination of genetic and clinical features [7], and eventually by the exclusion of other disorders that may mimic FSHD. Although in the classical form and in familial cases the diagnosis is often straightforward, more difficulties may arise with sporadic or atypical patients. In particular, the differential diagnosis with some forms of LGMDs with overlapping clinical features [8], [9] or congenital myopathies [10] can be sometimes challenging and additional supportive instrumental criteria may be needed to target the appropriate genetic investigations.

In this study, we propose an MRI protocol to study the muscles of the upper girdle. We applied this protocol to a large cohort of FSHD patients, in order to assess the pattern and degree of involvement in this disease and to compare the results with other myopathies also showing marked upper girdle involvement.

## Methods

### Patients

One hundred-eight consecutive FSHD patients meeting the criteria listed below (52 males and 56 females, mean age 41±15, mean EcoRI fragment length 25±6 kb) were enrolled in the study. Disease severity was evaluated with the Clinical Severity Score (CSS) scale [11]. In order to be enrolled in the study, two criteria had to be fulfilled:

- Patients had to belong to families with at least one member affected by FSHD with typical clinical features and molecular study showing a 4q35 BlnI resistant, p13-E11 EcoRI fragment shorter than 40 kb segregating with the disease, and autosomal dominant inheritance. Sporadic patients were admitted only if carrying a de-novo short fragment.
- Clinical involvement of scapular girdle (CSS ≥1).

Subjects with at least one of the following characteristics were excluded:

- Somatic mosaics for 4q35 shortened fragments.
- Non-penetrant gene carriers.
- Typical FSHD patients not carrying short fragments (FSHD2).
- Patients affected by concomitant systemic diseases, which could cause myopathic or neuropathic features with denervation.
- Patients bearing devices not compatible with MRI.

Demographic, clinical and genetic features of patients are reported in Tables S1 and S3. Enrolled patients were also asked to report their handedness.

Scans were also acquired from 45 patients affected by other genetic or sporadic myopathies with upper girdle involvement: 14 patients affected by LGMD2A, 5 by LGMD2B/Miyoshi myopathy (MM), 4 by LGMD1D, 4 by acid maltase deficiency – type 2 glycogen storage disease (AMD), 3 manifesting carriers of dystrophinopathy, 3 by hereditary myopathy with early respiratory failure (HMERF), 3 by LGMD2L, 2 by sporadic late onset nemaline myopathy (SLONM), 2 by *TPM2*-related myopathy, 2 by *MYH7*-related myopathy, 1 by nemaline myopathy, 1 *FLNC*-mutated myofibrillar myopathy, 1 by anti-signal recognition particle (SRP) myopathy.

This study was approved by the Ethics Committee of the Catholic University School of Medicine. All the involved subjects gave their written informed consent.

### MRI protocol

MRI studies were performed on a 1.5 Tesla equipment (Magnetom Espree, Siemens AG) using integrated matrix coils (phased array). Non-contrast axial, coronal and sagittal high-resolution images were obtained using T1-TSE (turbo spin echo) sequences (T1-W). Fat suppressed sequences (STIR) on the axial plane were also performed in order to assess muscle edema. Axial T1-TSE images (TR/TE of 400/13 ms, thickness/gap 4 mm/0, 4 mm, FOV 370 mm) were performed by two or three contiguous stacks (about 30 slices for each stack) to obtain an anatomic coverage from the skull base to the D10 vertebral body. STIR axial images (TR/TE/TI 3000/35/160 ms) were obtained with the same geometry and the same number of stacks of the T1 sequences. Coronal T1-TSE images (TR/TE of 450/13 ms, thickness/gap 3,5 mm/0,35 mm, FOV 400 mm) were performed in three separate acquisitions of about 25 slices for each stack with specific plane orientations for anterior thoracic muscles, posterior thoracic muscles and neck muscles. The anterior muscles were investigated by a series of slices oriented along the axis of the pectoralis major muscle. The best plane for the imaging of posterior dorsal muscles was parallel to the dorsal kyphosis. For the coronal imaging of the neck the stack was positioned along the major axis of the neck. Sagittal T1-TSE images (TR/TE of 624/13 ms, thickness/gap 5 mm/0,5 mm, FOV 400 mm) were obtained in order to cover the entire body from one shoulder to the other. The duration of the examination was approximately 45 minutes. Figure S1 shows the shoulder girdle muscles assessed with our protocol. The scans were acquired in the period September 2010-February 2013. Data can be made available to interested researchers upon request.

### Scoring system

Fourteen muscles or muscle groups were scored for each patient on each side. The serratus anterior was considered as a whole entity including its three components (superior, middle and inferior). Based on T1-W images, muscles were evaluated on all available sections, and a comprehensive score describing the degree of involvement was given according to the following scale:

• 0: unaffected muscle (normal signal on T1-W images).
• 1: definite abnormal hyperintense areas involving less than 50% of the muscle volume.
• 2: abnormal hyperintense areas involving more than 50% of the muscle volume but still with some clearly preserved areas.
• 3: muscle completely replaced by fatty fibrous tissue or atrophic.

The scoring was performed independently by two neurologists experienced in neuromuscular imaging (ER and GT). In case of discordance, agreement was reached by consultation. The complete set of data of muscle scores for FSHD and other myopathies is provided in Tables S1 and S2.

A T1-MRI score was calculated as the sum of the scores of involvement of the individual muscles in each patient. STIR sequences were evaluated assessing the presence or absence of signal hyperintensity in each muscle.

### Statistical analysis

Data were analyzed using SPSS Statistics ver.18. All p-values were two-sided, and p<0.05 was considered statistically significant. Shapiro-Wilk test was used to assess data distribution, and each time the appropriate statistic was chosen accordingly. Global and per muscle asymmetric involvement between right and left side was tested using the Wilcoxon signed rank test and the sign test respectively. The Wilcoxon-Mann-Whitney test was used to verify T1-MRI score and age differences between FSHD and LGMD groups. Correlation between CSS and T1-MRI score was tested using Spearman's rho. A multiple linear regression model was built to test the association between age, sex, and EcoRI fragment length (independent variables) and T1-MRI score (dependent variable). A square root transformation of the T1-MRI score was applied to stabilize the variance and to obtain approximately normally distributed residuals. Fisher's exact test was used to explore influence of handedness on T1-MRI score side prevalence.

## Results

### Muscle involvement in FSHD

None of the recruited patients had a normal MRI. The trapezius was the most frequently involved muscle: it was affected on at least one side in all of the patients, and it was the only affected muscle in 12/108 (11%). In 11 of these 12 patients there was no clinical involvement of lower limbs (CSS ≤2), and one had only an impaired ankle dorsiflexion (CSS 2.5). In 87% of patients the trapezius was also the most severely affected muscle on MRI. The second most frequently affected muscle was the serratus anterior (92 out of 108 patients, 85%). Trapezius and serratus anterior were the only muscles affected in 6 patients. Other commonly affected muscles were the latissimus dorsi (at least one muscle involved in 77% of patients) and pectoralis major (75%).

**Figure 1:**
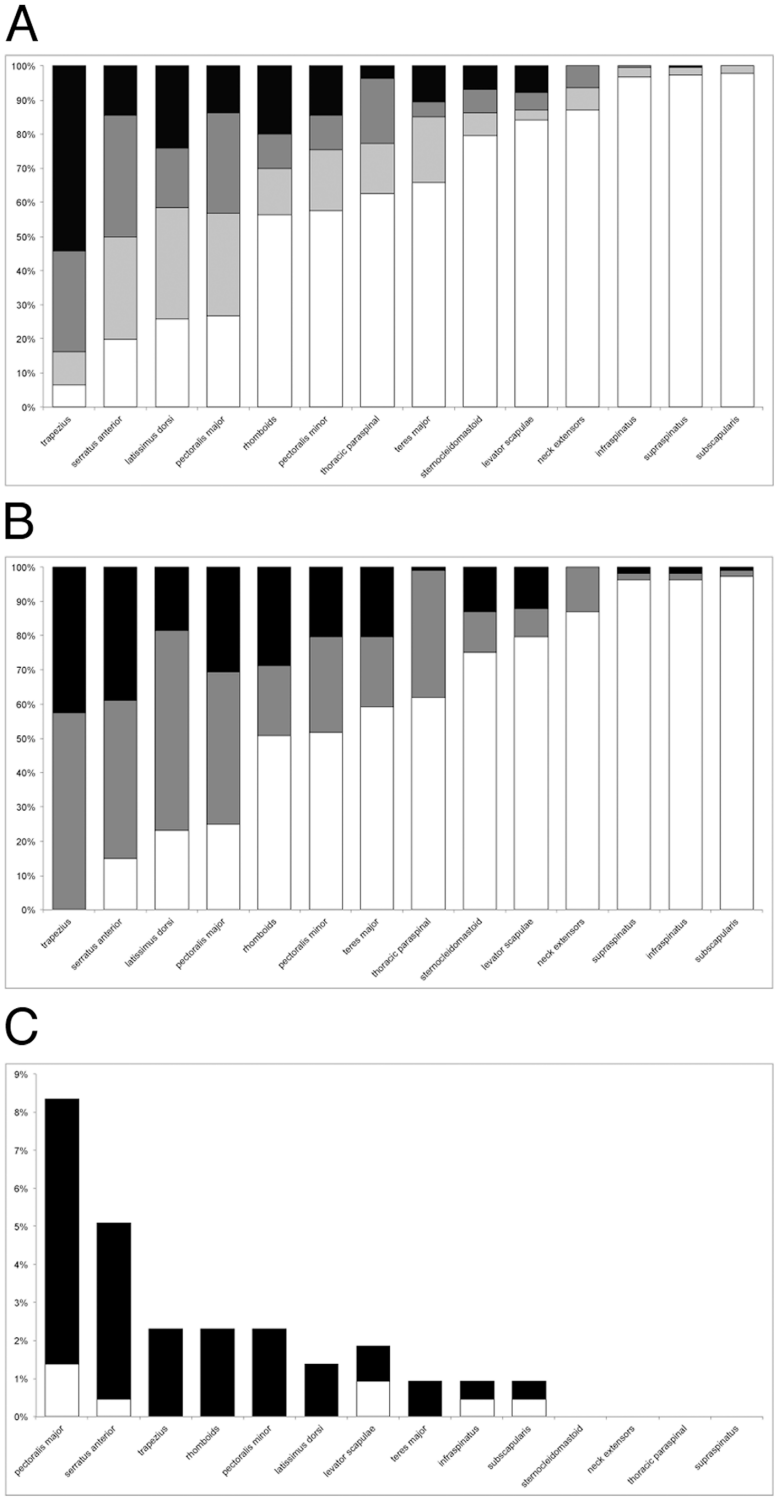
Summary of muscle involvement in FSHD. (A) Percentage of involvement of the different muscles. White columns: unaffected; light gray columns: affected with score 1; dark gray columns: affected with score 2; black columns: affected with score 3. (B) Frequency of symmetrical and asymmetrical involvement of individual muscles across all the patients. White columns: unaffected; gray columns: affected symmetrically; black columns: affected asymmetrically. (C) Percentage of involvement on STIR sequences of T1-W normal (white) or abnormal (black) muscles.

**Figure 2:**
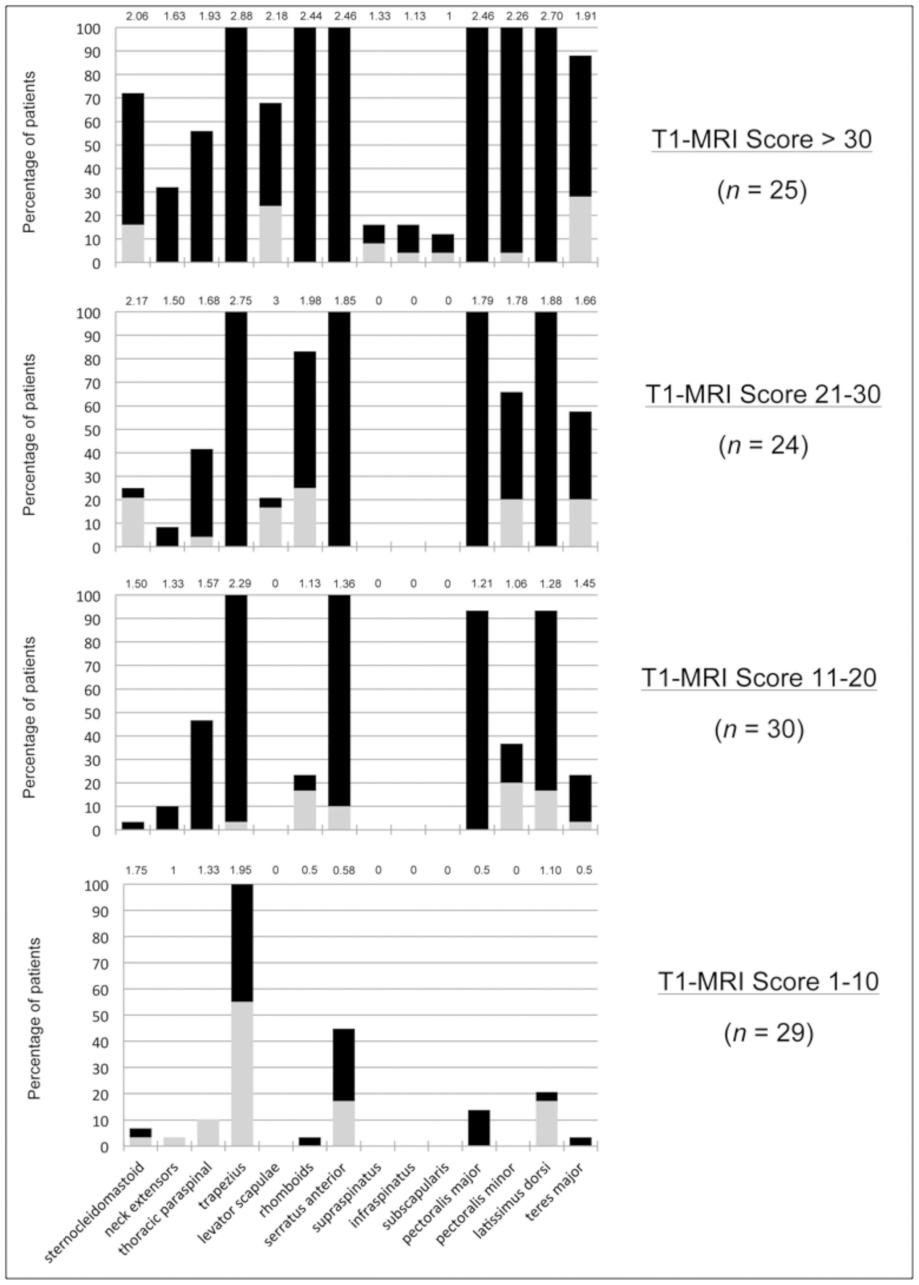
Graphical representation of the distribution of muscle involvement across different disease severities. Patients are subdivided into 4 categories of severity according to the T1-MRI score values. The contribution of the progressive involvement of the different muscles to the T1-MRI score is represented as percentage of patients in whom each muscle is affected monolaterally (gray column) or bilaterally (black column) and mean score for each muscle (number on top of the columns).

The involvement of the other muscles was more variable (Figure 1, Figure 2 and Table S1). The levator scapulae was involved only in patients with moderate or advanced disease, never in patients without lower limb clinical involvement (CSS ≤2), and it was generally severely affected (score 1 six times, 2 eleven times and 3 seventeen times).

In contrast, the subscapularis was the most spared muscle (affected in only 3 out of 108 patients, always with a score of 1). Other muscles that were commonly spared were the supraspinatus and infraspinatus (affected in 4/108 patients). These muscles were involved only in the most severe patients: no patient with EcoRI fragment >21 kb had involvement of these muscles, and their score reached a value of 3 only once in the patient with the shortest fragment in our cohort (10 kb).

**Figure 3:**
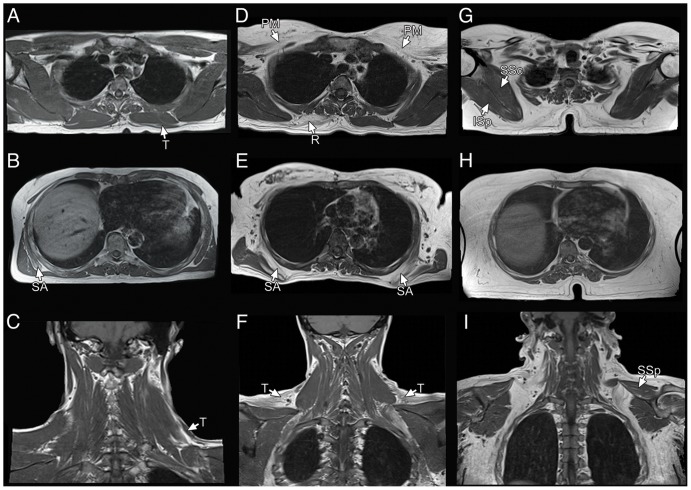
T1-W MR images in FSHD patients with different disease severity. In patients with initial disease (A–C) the involvement is generally restricted to the trapezius and serratus anterior muscles, often asymmetrically. In moderately affected patients (D–F), a frequent combination is constituted by bilateral involvement of the trapezius, serratus anterior, pectoralis major and asymmetric rhomboids involvement. In more advanced disease (G–I), other muscles become involved but still with typical complete sparing of the supraspinatus, infraspinatus and subscapularis. T: trapezius; SA: serratus anterior; PM: pectoralis major; R: rhomboids; SSp: supraspinatus; ISp: infraspinatus; SSc: subscapularis.

In order to identify a general pattern of involvement, we explored the most frequent combinations of involvement of different muscles. As a result, we found that:

- 85% of FSHD patients had a combined involvement of at least one trapezius and one serratus anterior;
- 74% had a combined involvement of at least one trapezius, one serratus anterior and one latissimus dorsi;
- 71% had a combined involvement of at least one trapezius, one serratus anterior, one latissimus dorsi and one pectoralis major;
- 66% had a combined involvement of at least one trapezius, one serratus anterior, one latissimus dorsi, one pectoralis major and a complete sparing of both subscapularis, supra and infraspinati muscles (Figure 3).

To get additional clues useful for diagnostic purposes, we also compared the severity of involvement of muscle couples among the most affected muscles, and found that trapezius was at least as affected as serratus anterior in 96% cases and latissimus dorsi was at least as affected as teres major in 95% cases.

### Asymmetry

**Figure 4:**
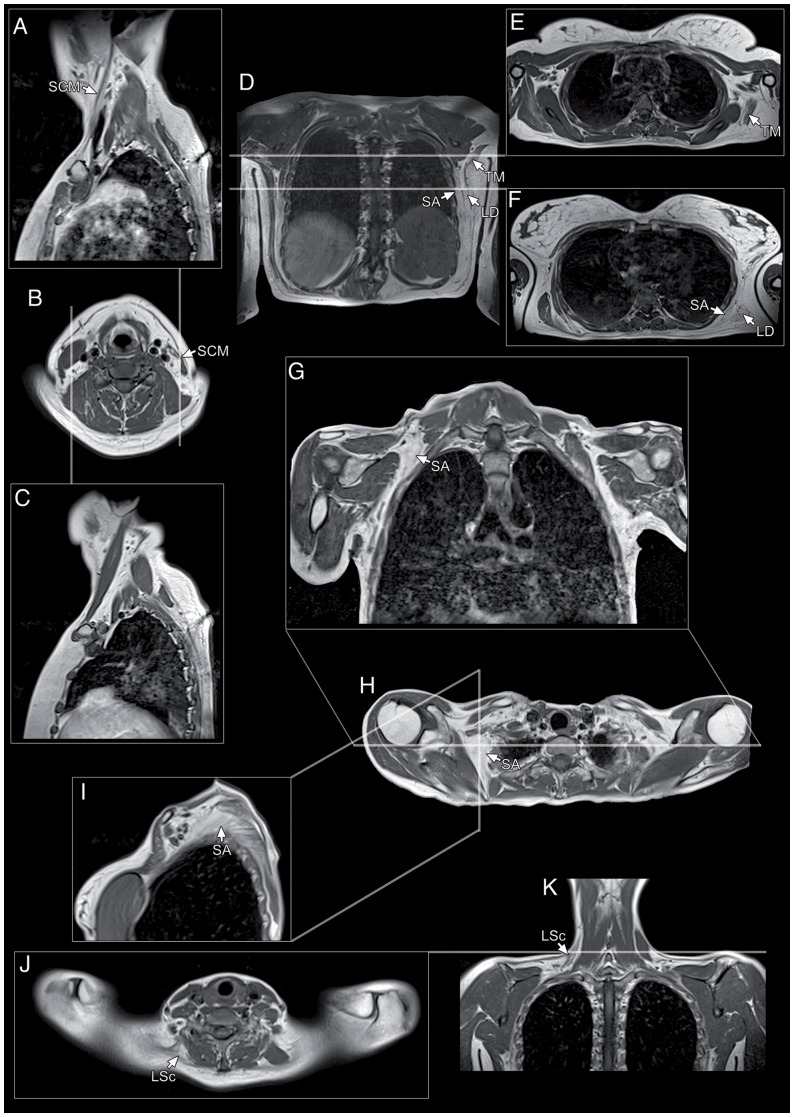
Identification of asymmetrical muscle involvement in FSHD using different projections. (A–C) Monolateral atrophy of sternocleidomastoid muscle evident on sagittal and axial sections. Sagittal planes of sectioning used in our protocol were particularly useful to identify and to follow muscles across all their length thus providing a comprehensive assessment on their degree of involvement. (D–F) Coronal section (D) and two axial sections at different levels (E–F) showing asymmetric replacement of the serratus anterior, teres major and latissimus dorsi on one side. (G–I) Involvement of the superior portion of the right serratus anterior can be appreciated in all the three projections used. (J–K) Asymmetric replacement of levator scapulae with sparing of the contralateral on axial and corresponding coronal section. SCM: sternocleidomastoid; SA: serratus anterior; TM: teres major, LD: latissimus dorsi; LSc: levator scapulae.

In 89% of the patients (96 out of 108) there was an asymmetrical involvement of at least one muscle, considering a side-to-side difference of at least one point in the 0–3 scale, whilst 47% of patients had a differential involvement of at least two points. Sixty-one % of patients had at least one affected muscle with sparing of the contralateral and 37% had one severely or totally fatty-replaced muscle (score 2–3) whilst the contralateral was completely spared. The muscles most frequently showing asymmetries were: trapezius (in 43% patients), serratus anterior (39%), pectoralis major (31%) and rhomboids (29%) (Figure 1, Figure 4, and Table S4).

The overall involvement was higher on the right side in 63 patients (60 right-handed and 3 left-handed), versus 24 with predominant involvement on the left side (20 right-handed and 4 left-handed), with a significant side-to-side difference in the T1-MRI scores (i.e., the sums of the scores of the individual muscles in each patient) of each side (p<0.001) (Table S3). A non-significant influence of handedness on the overall side prevalence was reported (p = 0.10).

### STIR hyperintensities

**Figure 5:**
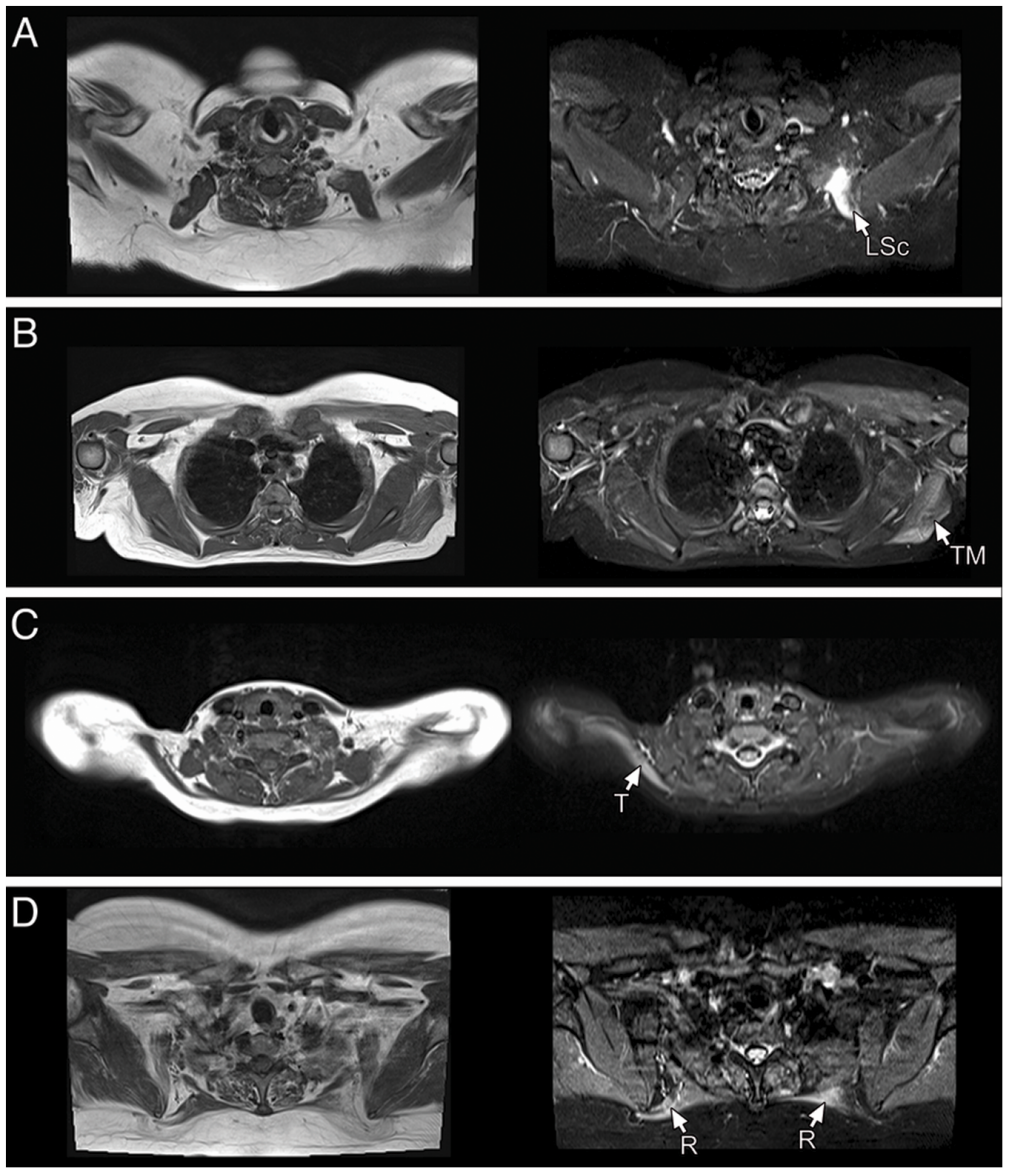
T1-W images (left) with corresponding STIR images (right). STIR hyperintensities can be detected in different muscles in absence or with different degree of abnormalities on T1-W images. (A): levator scapulae. (B): teres major. (C) trapezius. (D) rhomboids.

Thirty-four patients (31%) had at least one muscle hyperintense on STIR sequences. Overall, 2% of the muscles showed abnormal signal on STIR sequences. The muscles more frequently displaying these abnormalities were the pectoralis major (18/216 muscles, 8%), serratus anterior (5%), trapezius, rhomboids and pectoralis minor (2%). In one fourth of the cases (0,26% of the total of the muscles), STIR hyperintensities were found in muscles displaying normal signal on T1-W images (Figures 1 and 5).

### Effect of gender, age and genetics on upper girdle imaging involvement

A strong correlation between T1-MRI score and CSS was found (Spearman's rho  = 0.76, p<0.001). The multiple linear regression model built including all the independent variables tested in our population showed that age and EcoRI fragment length had a significant independent influence (regression coefficient 0.04 and −0.09 respectively with p<0.001) on the root transformed T1-MRI score. In contrast T1-MRI score was not significantly influenced by sex differences even though a protective effect of the female gender was suggested (coefficient −0.43, p = 0.192). Less than 20% of the overall variability of the T1-MRI score could be explained by our model (Table S3).

### Comparison with other diseases

**Figure 6:**
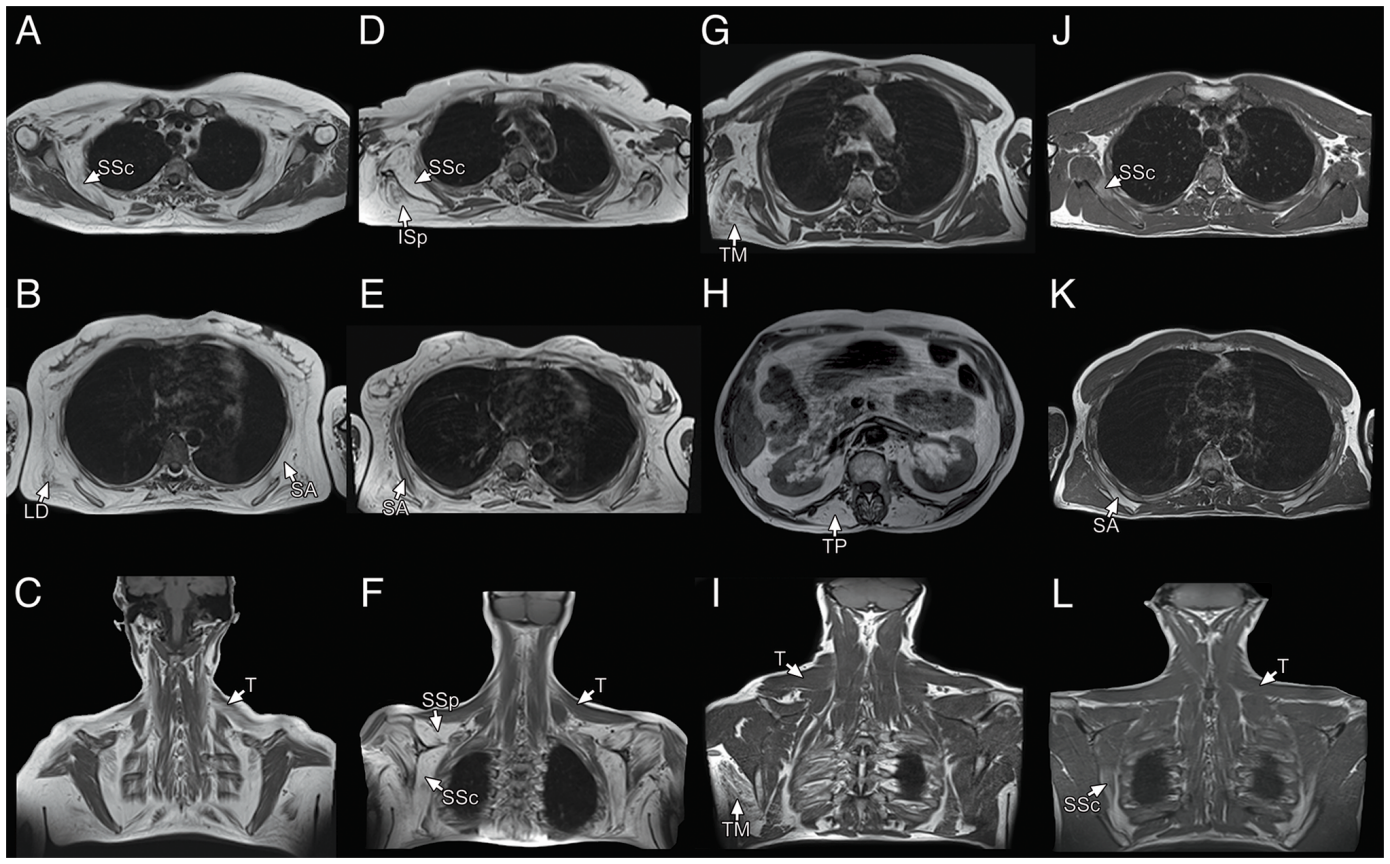
Examples of upper girdle imaging involvement in other myopathies. LGMD2A (A–C): prominent and symmetric involvement of the serratus anterior, latissimus dorsi and subscapularis muscles with partial sparing of the trapezius. LGMD2B (D–F) major involvement of the supraspinatus, infraspinatus and subscapularis muscles, with relative sparing of the trapezius and serratus anterior. LGMD2L (G–I): isolated involvement of one teres major and thoracic paraspinal muscles, without replacement of trapezius muscles. LGMD1D (J–L): mild involvement of the serratus anterior and subscapularis muscles, again with complete sparing of trapezius. SA: serratus anterior; LD: latissimus dorsi; SSc: subscapularis; T: trapezius; SSp: supraspinatus; ISp: infraspinatus; TM: teres major; TP: thoracic paraspinal.

The pattern detected in FSHD (i.e., trapezius involvement later followed by serratus anterior, latissimus dorsi and pectoralis major, with sparing of spinati and subscapularis muscles) was not found in any of the patients with other muscle diseases, with the exception of one AMD patient who showed an isolated trapezius involvement compatible with initial FSHD. The same pattern was not observed in the other AMD patients, who showed a prominent subscapularis involvement together with thoracic paraspinal muscles that could rule out FSHD. In LGMD2A, the most frequently and heavily affected muscles were teres major, serratus anterior and pectoralis major. The subscapularis was always affected on both sides, often severely. The spinati muscles were also quite invariably affected, often to a degree comparable with trapezius and serratus anterior. The trapezius was more involved than serratus anterior only in one patient. In LGMD2B/MM patients, the most affected muscles were teres major, subscapularis, supraspinatus, infraspinatus and pectoralis major. The levator scapulae was usually completely spared; trapezius and serratus anterior were relatively spared as well. Overall involvement was higher (p = 0.009) in LGMD2A and LGMD2B/MM compared with FSHD, without significant age differences (p>0.5). Fewer patients could be examined for each of the other genetic or acquired myopathies (Figure 6 and Table S2 legend for details). In the control group both asymmetry and STIR hyperintensities were significantly less frequent than in FSHD (Table S4).

## Discussion

In FSHD, upper girdle involvement on imaging had been to date assessed only in a small cohort using computed tomography [12]. The use of MRI that has the advantage of multiple slices and sequences and better anatomical details, allowed us a more systematic evaluation of individual muscles, providing data that so far have relied only on clinical examinations with non-univocal results. The most comprehensive of these surveys is G. Padberg's thesis [13], in which the author describes the rhomboids as the weakest muscles, followed by lower trapezius, pectoralis major and serratus anterior, with upper trapezius being involved only later in the disease. Although supraspinatus and infraspinatus were described as spared by Landouzy and Dejerine [14], most of the subsequent authors noticed weakness and wasting of these muscles [13]. However, clinical assessment is hampered by the fact that not every upper girdle muscle can be individually explored by manual muscle testing and evaluation of each muscle may be not reliable since it depends on the good function of the other scapular fixators [13]. Therefore, whilst correctly localizing the impaired function can be relatively straightforward when single muscles are completely defective such as in nerve injuries [15], difficulties may arise when combinations of total and partial muscle defects are involved [16].

Using MRI we were able to better characterize the involvement of the upper girdle muscles in a large cohort of consecutive FSHD patients with a broad spectrum of clinical severity. With the limitations of a cross-sectional study, we can however hypothesize that the different frequencies of involvement may mirror the sequential involvement of the different muscles, whose assessment is a key issue for understanding the natural history of the disease (Figure 2). The trapezius and to a slightly lesser extent the serratus anterior are the earliest involved and the most affected muscles, and isolated trapezius involvement is usually present in absence of clinically evident impairment in lower limbs. The latissimus dorsi and pectoralis major are also frequently involved. On the contrary, the subscapularis and spinati muscles are typically spared until advanced disease stages.

The severity on upper girdle imaging (quantified as T1-MRI score) was significantly correlated with overall clinical severity, and influenced by age and EcoRI fragment length. Although not reaching statistical significance, a trend towards a protective effect of female gender on disease severity is suggested by our multiple linear regression analysis. The effect of gender differences on disease penetrance [17], [18] was not explored in our study in which only symptomatic patients were imaged. Notably, the statistical model developed considering sex, age and fragment length together is not able to accurately predict the T1-MRI score, thus implying a role for other potential modifiers.

We also confirmed that asymmetry is another very common feature in FSHD. Interestingly, we found a significant prevalence of involvement on the right side. This is in accordance with previous findings and has been put in relation with mechanical factors and in particular with preferential use of the right side by right-handed people [19]. Although we cannot confirm an influence of handedness on side prevalence, this preferential involvement of the right side was indeed more evident for muscles actively participating to the scapular and upper limb movements (such as serratus anterior, pectoralis, and trapezius) and less evident for long, axial muscles (Table S3).

The use of a structured protocol including STIR sequences also provided some clues to understand the pathogenesis of the disease, confirming that hyperintensities on STIR sequences are a feature of FSHD and can be found also in upper limb muscles. Since they are present in muscles affected in early stage and/or not yet showing signs of fatty replacement, STIR changes may represent an early marker of involvement and disease activity [20], [21]. We have already hypothesized a potential pathogenic role of the inflammatory process identified by STIR hyperintensities in the development and progression of the disease [20], [22]. The minor frequency in the upper girdle muscles compared to what is reported in lower limbs in this disease [23]–[27] could be explained either with a different duration of the process at single muscle level, i.e. STIR hyperintensities might be less long-lasting in upper girdle than lower limb muscles, or with a different timing of the disease itself, i.e. upper girdle muscles might be affected by STIR abnormalities very early in the disease course thus making these alterations less likely to be detected in a scan of an already symptomatic patient, or with a combination of both. Further evidences are required to clarify whether STIR hyperintensities mark a place of “active” inflammation, eventually driven by the ectopic expression of *DUX4* [28], and whether they could represent a therapeutic target.

In order to establish whether the pattern of upper girdle muscle impairment observed in FSHD was common to other forms, we also evaluated scans from patients with myopathies characterized by upper girdle weakness and/or scapular winging. As the number of scans available for each of these forms was limited we were unable to provide a comprehensive overview of upper girdle imaging in each of the other myopathies but we found that the pattern in FSHD patients was rather distinct since, with one partial exception, it was not present in any of the other muscle disorders. Even if the number were small, we observed that LGMD2A and 2B had an overall more severe involvement and relative sparing of the trapezius, with invariable involvement of the subscapularis and often spinati muscles [29]. Trapezius was also spared in the autosomal dominant LGMD1D, in which the scapular winging [30] was associated with serratus anterior involvement. Finally, in agreement with previous studies, axial muscles and in particular long thoracic muscles in *TPM2*-mutated patients [31] were predominantly involved in the adult congenital myopathy patients studied with our protocol and, together with subscapularis, also in AMD [32], [33]. Asymmetries, common in FSHD, were less common the control cohort with the exception of female carriers of dystrophinopathy [1] and LGMD2L [34], but the patterns of upper girdle involvement are clearly different between these diseases and FSHD (Table S2). Further data on upper girdle imaging of other myopathies is needed to better characterize their spectrum and pattern, which appears to be characteristic for some of them, and to confirm that there are no overlaps with FSHD.

In conclusion, this study suggests that the proposed protocol allows a reliable identification and evaluation of the involvement of the most significant muscles and muscle groups responsible for clinical symptoms in FSHD, including the thin and longitudinally oriented ones, with adequate resolution and anatomical coverage. In FSHD diagnostic workup, our imaging protocol is suitable when the available MRI equipment, even if characterized by high performance, does not allow complete whole-body examinations with coverage from head to feet. We used an integrated matrix coil (phased array) instead of an embedded body coil, as we preferred to obtain high-resolution images despite a slight lengthening in the time of examination. The use of three different coronal planes, for the anterior and posterior thoracic musculature and for neck muscles, as well as of the sagittal planes, highly improved the detail on the muscles of these areas that have particular anatomic features and spatial orientations related to the different conformations of the thoracic wall (Figure 4). These differences are more pronounced in myopathic patients who often display anatomical modifications of thoracic wall due to muscle function impairment. However, the acquisitions of scans without sagittal sections and with only one coronal plane could allow a faster examination without loss of important information.

At variance with what observed using lower limb MRI [37], we found a consistent pattern of involvement in this disease. These results suggest that upper girdle imaging may be used as a diagnostic instrument for FSHD, for instance to clarify families in which a permissive genetic background is segregating but members do not show a typical phenotype. The use of upper girdle MRI can also be an additional tool for correctly assessing patients' motor function, planning surgical treatments and rehabilitation protocols in FSHD.

A partial limitation of our study is the absence of quantitative measurements of both fat content and muscle edema with dedicated techniques such as fat infiltration fraction, T2-mapping, DWI and DTI, and MR spectroscopy [35], [36]. Acquisition of scans with such techniques could be particularly useful in a less clinically-oriented, diagnostic context but for instance in longitudinal natural history studies, where more objective and sensitive measurements are of key importance. However, the pattern of involvement and the frequency distribution allowed detecting the muscles that are involved in the early phases of the disorder and provides a baseline for the assessment of sequential involvement over time. The correct identification of the muscles affected in early phases and the knowledge of the muscles most likely to show changes are also eventually important in the design of clinical trials since this knowledge has implications for the choice of targets in the screening of potential therapies and to stratify patients to be enrolled. Moreover, MRI presents some features, such as being a non-invasive, harmless method to evaluate subtle changes in absence of weakness, which might make it an ideal biomarker in clinical trials [27]. Longitudinal studies on large cohorts are needed to fully understand the natural history of the disease and to delineate the evolution of the STIR changes.

## Supporting Information

Figure S1 **Normal anatomy of shoulder girdle muscles visualized with our protocol on different plans of sectioning.** ISp: infraspinatus; LD: latissimus dorsi; LSc: levator scapulae; NE: neck extensors; PM: pectoralis major; Pm: pectoralis minor; R: rhomboids; SA: serratus anterior; SCM: sternocleidomastoid; SSc: subscapularis; SSp: supraspinatus; T: trapezius; TM: teres major; TP: thoracic paraspinal.
(TIF)

Table S1 **Summary of individual muscle scores across all FSHD patients, progressively ordered by T1-MRI score.**
(DOCX)

Table S2 **Summary of individual muscle scores across all the non-FSHD patients, subdivided by diagnosis.** In LGMD2L asymmetric involvement is a feature, but the teres major is the most involved muscle, followed by thoracic paraspinal muscles with relative sparing of trapezius and serratus anterior. In LGMD1D, the serratus anterior is always mildly involved, sometimes together with spinati or subscapularis, and the trapezius is always spared. In manifesting female carriers of dystrophinopathy, asymmetrical involvement is present together with trapezius and serratus anterior involvement, but heavy and early involvement of supra- and infraspinatus, pectoralis minor and subscapularis help in the differential diagnosis with FSHD. In HMERF, the sternocleidomastoid is always affected and the trapezius is always spared. In SLONM, serratus anterior and subscapularis are more affected than trapezius. In *MYH7*-related myopathy, the sternocleidomastoid is heavily affected in the more severe patient and subscapularis involvement would make the diagnosis of FSHD unlikely in the milder one. In *TPM2*-related myopathy, the trapezius is always spared and thoracic paraspinal and neck extensors are affected. STIR hyperintensities could be found only in a minority of non-FSHD patients. Differences between LGMD2A, LGMD2B, AMD and FSHD patterns are discussed in the text.
(DOCX)

Table S3 **Summary of statistical analyses.**
(DOCX)

Table S4 **Asymmetric involvement and STIR hyperintensities in FSHD and other myopathies.**
(DOCX)

## References

[1] Tasca G, Monforte M, Iannaccone E, Laschena F, Ottaviani P, et al (2012) Muscle MRI in female carriers of dystrophinopathy. Eur J Neurol 19: 1256–1260. 22583668 10.1111/j.1468-1331.2012.03753.x

[2] Mercuri E, Pichiecchio A, Allsop J, Messina S, Pane M, et al (2007) Muscle MRI in inherited neuromuscular disorders: past, present, and future. J Magn Reson Imaging 25: 433–440. 17260395 10.1002/jmri.20804

[3] Wattjes MP, Kley RA, Fischer D (2010) Neuromuscular imaging in inherited muscle diseases. Eur Radiol 20: 2447–2460. 20422195 10.1007/s00330-010-1799-2 PMC2940021

[4] Padberg GW, van Engelen BG (2009) Facioscapulohumeral muscular dystrophy. Curr Opin Neurol 22: 539–542. 19724227 10.1097/WCO.0b013e328330a572

[5] Lemmers RJ, van der Vliet PJ, Klooster R, Sacconi S, Camano P, et al (2010) A unifying genetic model for facioscapulohumeral muscular dystrophy. Science 329: 1650–1653. 20724583 10.1126/science.1189044 PMC4677822

[6] Scionti I, Greco F, Ricci G, Govi M, Arashiro P, et al (2012) Large-scale population analysis challenges the current criteria for the molecular diagnosis of fascioscapulohumeral muscular dystrophy. Am J Hum Genet 90: 628–635. 22482803 10.1016/j.ajhg.2012.02.019 PMC3322229

[7] Lemmers RJ, O'Shea S, Padberg GW, Lunt PW, van der Maarel SM (2012) Best practice guidelines on genetic diagnostics of Facioscapulohumeral muscular dystrophy: workshop 9th June 2010, LUMC, Leiden, The Netherlands. Neuromuscul Disord 22: 463–470. 22177830 10.1016/j.nmd.2011.09.004

[8] Sacconi S, Camano P, de Greef JC, Lemmers RJ, Salviati L, et al (2012) Patients with a phenotype consistent with facioscapulohumeral muscular dystrophy display genetic and epigenetic heterogeneity. J Med Genet 49: 41–46. 21984748 10.1136/jmedgenet-2011-100101 PMC3560331

[9] Leidenroth A, Sorte HS, Gilfillan G, Ehrlich M, Lyle R, et al (2012) Diagnosis by sequencing: correction of misdiagnosis from FSHD2 to LGMD2A by whole-exome analysis. Eur J Hum Genet 20: 999–1003. 22378277 10.1038/ejhg.2012.42 PMC3421126

[10] Felice KJ, Grunnet ML (1997) Autosomal dominant centronuclear myopathy: report of a new family with clinical features simulating facioscapulohumeral syndrome. Muscle Nerve 20: 1194–1196. 9270681 10.1002/(sici)1097-4598(199709)20:9<1194::aid-mus19>3.0.co;2-t

[11] Ricci E, Galluzzi G, Deidda G, Cacurri S, Colantoni L, et al (1999) Progress in the molecular diagnosis of facioscapulohumeral muscular dystrophy and correlation between the number of KpnI repeats at the 4q35 locus and clinical phenotype. Ann Neurol 45: 751–757. 10360767 10.1002/1531-8249(199906)45:6<751::aid-ana9>3.0.co;2-m

[12] Wang CH, Leung M, Liang WC, Hsieh TJ, Chen TH, et al (2012) Correlation between muscle involvement, phenotype and D4Z4 fragment size in facioscapulohumeral muscular dystrophy. Neuromuscul Disord 22: 331–338. 22153988 10.1016/j.nmd.2011.10.018

[13] Padberg GW (1982) Facioscapulohumeral disease. Thesis. Leiden, The Netherlands: Leiden University press.

[14] Landouzy L, Dejerine J (1885) De la myopathie atrophique progressive. Revue de Médecine 5: 81–117.

[15] Martin RM, Fish DE (2008) Scapular winging: anatomical review, diagnosis, and treatments. Curr Rev Musculoskelet Med 1: 1–11. 19468892 10.1007/s12178-007-9000-5 PMC2684151

[16] Monforte M, Ricci E, Iannaccone E, Tasca G (2013) Teaching Video NeuroImages: Complicated scapular winging. Neurology 81: e95. 24042578 10.1212/WNL.0b013e3182a4a4e6

[17] Tonini MM, Passos-Bueno MR, Cerqueira A, Matioli SR, Pavanello R, et al (2004) Asymptomatic carriers and gender differences in facioscapulohumeral muscular dystrophy (FSHD). Neuromuscul Disord 14: 33–38. 14659410 10.1016/j.nmd.2003.07.001

[18] Zatz M, Marie SK, Cerqueira A, Vainzof M, Pavanello RC, et al (1998) The facioscapulohumeral muscular dystrophy (FSHD1) gene affects males more severely and more frequently than females. Am J Med Genet 77: 155–161. 9605290

[19] Brouwer OF, Padberg GW, van der Ploeg RJ, Ruys CJ, Brand R (1992) The influence of handedness on the distribution of muscular weakness of the arm in facioscapulohumeral muscular dystrophy. Brain 115: 1587–1598. 1422805 10.1093/brain/115.5.1587

[20] Tasca G, Pescatori M, Monforte M, Mirabella M, Iannaccone E, et al (2012) Different molecular signatures in magnetic resonance imaging-staged facioscapulohumeral muscular dystrophy muscles. PLoS One 7: e38779. 22719944 10.1371/journal.pone.0038779 PMC3374833

[21] Friedman SD, Poliachik SL, Otto RK, Carter GT, Budech CB, et al (2013) Longitudinal features of stir bright signal in FSHD. Muscle Nerve doi: 10.1002/mus.23911 10.1002/mus.23911 23720194

[22] Frisullo G, Frusciante R, Nociti V, Tasca G, Renna R, et al (2011) CD8(+) T cells in facioscapulohumeral muscular dystrophy patients with inflammatory features at muscle MRI. J Clin Immunol 31: 155–166. 21063901 10.1007/s10875-010-9474-6

[23] Tasca G, Monforte M, Iannaccone E, Frusciante R, Laschena F, et al (2011) P2.38 Lower limb muscle MRI in a large cohort of FSHD patients. Neuromuscular Disorders 21: 671.

[24] Straub V, Carlier PG, Mercuri E (2012) TREAT-NMD workshop: pattern recognition in genetic muscle diseases using muscle MRI: 25–26 February 2011, Rome, Italy. Neuromuscul Disord 22 Suppl 2: S42–53. 22980768 10.1016/j.nmd.2012.08.002

[25] Kan HE, Scheenen TW, Wohlgemuth M, Klomp DW, van Loosbroek-Wagenmans I, et al (2009) Quantitative MR imaging of individual muscle involvement in facioscapulohumeral muscular dystrophy. Neuromuscul Disord 19: 357–362. 19329315 10.1016/j.nmd.2009.02.009

[26] Friedman SD, Poliachik SL, Carter GT, Budech CB, Bird TD, et al (2012) The magnetic resonance imaging spectrum of facioscapulohumeral muscular dystrophy. Muscle Nerve 45: 500–506. 22431082 10.1002/mus.22342

[27] Tawil R, Shaw DW, van der Maarel SM, Tapscott SJ (2014) Clinical trial preparedness in facioscapulohumeral dystrophy: Outcome measures and patient access: 8–9 April 2013, Leiden, The Netherlands. Neuromuscul Disord 24: 79–85. 24011701 10.1016/j.nmd.2013.07.009

[28] Geng LN, Yao Z, Snider L, Fong AP, Cech JN, et al (2012) DUX4 Activates Germline Genes, Retroelements, and Immune Mediators: Implications for Facioscapulohumeral Dystrophy. Dev Cell 22: 38–51. 22209328 10.1016/j.devcel.2011.11.013 PMC3264808

[29] Kesper K, Kornblum C, Reimann J, Lutterbey G, Schroder R, et al (2009) Pattern of skeletal muscle involvement in primary dysferlinopathies: a whole-body 3.0-T magnetic resonance imaging study. Acta Neurol Scand 120: 111–118. 19154541 10.1111/j.1600-0404.2008.01129.x

[30] Sarparanta J, Jonson PH, Golzio C, Sandell S, Luque H, et al (2012) Mutations affecting the cytoplasmic functions of the co-chaperone DNAJB6 cause limb-girdle muscular dystrophy. Nat Genet 44: 450–5, S1–2. 22366786 10.1038/ng.1103 PMC3315599

[31] Jarraya M, Quijano-Roy S, Monnier N, Behin A, Avila-Smirnov D, et al (2012) Whole-Body muscle MRI in a series of patients with congenital myopathy related to TPM2 gene mutations. Neuromuscul Disord 22 Suppl 2: S137–47. 22980765 10.1016/j.nmd.2012.06.347

[32] Carlier RY, Laforet P, Wary C, Mompoint D, Laloui K, et al (2011) Whole-body muscle MRI in 20 patients suffering from late onset Pompe disease: Involvement patterns. Neuromuscul Disord 21: 791–799. 21803581 10.1016/j.nmd.2011.06.748

[33] Alejaldre A, Diaz-Manera J, Ravaglia S, Tibaldi EC, D'Amore F, et al (2012) Trunk muscle involvement in late-onset Pompe disease: study of thirty patients. Neuromuscul Disord 22 Suppl 2: S148–54. 22980766 10.1016/j.nmd.2012.05.011

[34] Sarkozy A, Deschauer M, Carlier RY, Schrank B, Seeger J, et al (2012) Muscle MRI findings in limb girdle muscular dystrophy type 2L. Neuromuscul Disord 22 Suppl 2: S122–9. 22980763 10.1016/j.nmd.2012.05.012

[35] Janssen BH, Voet NB, Nabuurs CI, Kan HE, de Rooy JW, et al (2014) Distinct disease phases in muscles of facioscapulohumeral dystrophy patients identified by MR detected fat infiltration. PLoS One 9: e85416. 24454861 10.1371/journal.pone.0085416 PMC3891814

[36] Esposito A, Campana L, Palmisano A, De Cobelli F, Canu T, et al (2013) Magnetic resonance imaging at 7T reveals common events in age-related sarcopenia and in the homeostatic response to muscle sterile injury. PLoS One 8: e59308. 23555016 10.1371/journal.pone.0059308 PMC3595251

[37] Olsen DB, Gideon P, Jeppesen TD, Vissing J (2006) Leg muscle involvement in facioscapulohumeral muscular dystrophy assessed by MRI. J Neurol 253: 1437–1441. 16773269 10.1007/s00415-006-0230-z

