# Supplementary figures and images for: Upper Girdle Imaging in Facioscapulohumeral Muscular Dystrophy
Source: PLoS One. 2014 Jun 16;9(6):e100292. doi: 10.1371/journal.pone.0100292 (PMC4059711; doi:10.1371/journal.pone.0100292)

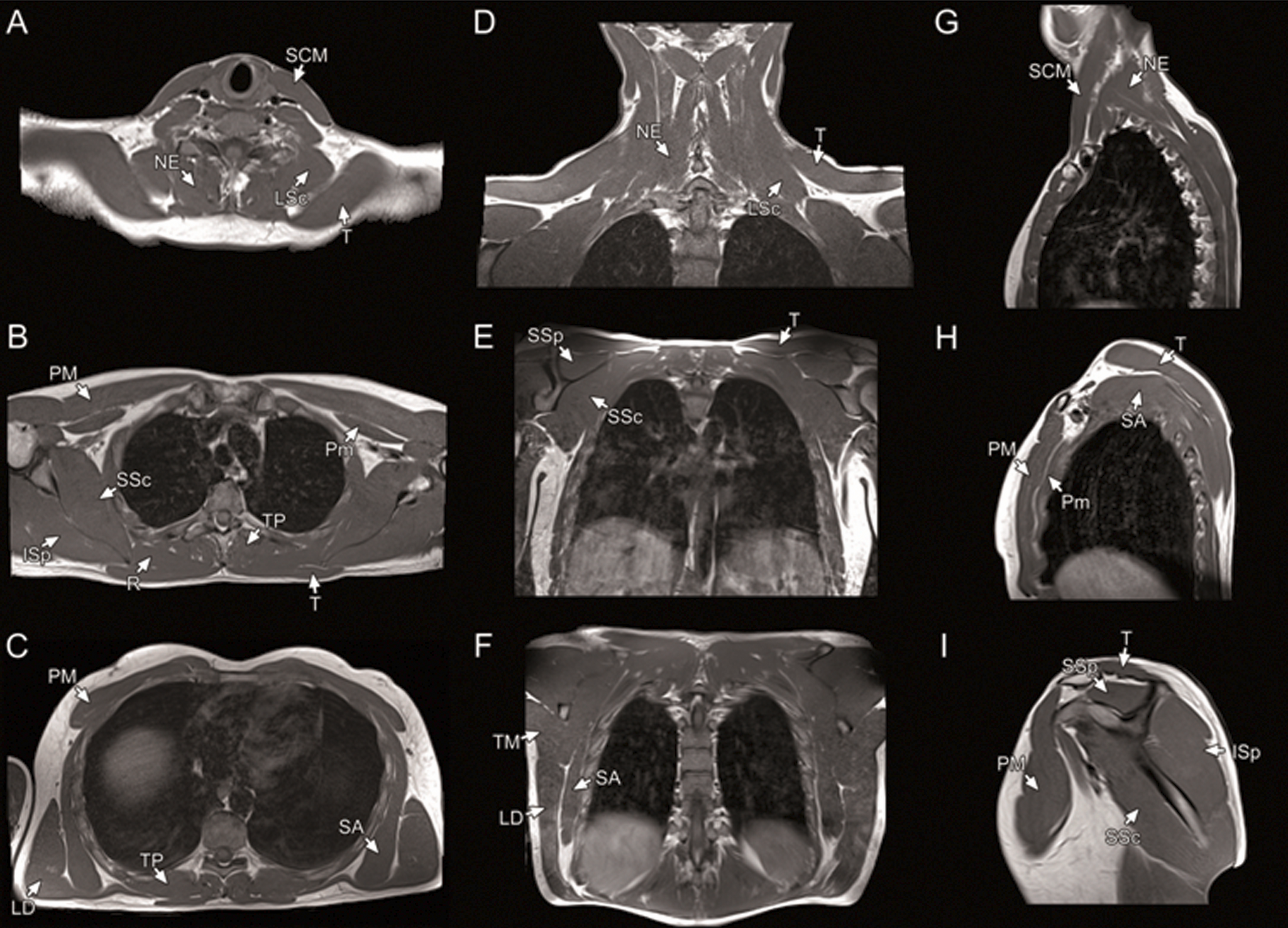

Supplement: Figure S1 — Normal anatomy of shoulder girdle muscles visualized with our protocol on different plans of sectioning. ISp: infraspinatus; LD: latissimus dorsi; LSc: levator scapulae; NE: neck extensors; PM: pectoralis major; Pm: pectoralis minor; R: rhomboids; SA: serratus anterior; SCM: sternocleidomastoid; SSc: subscapularis; SSp: supraspinatus; T: trapezius; TM: teres major; TP: thoracic paraspinal. (TIF) [file pone.0100292.s001.tif]
