# Supplementary material for: Upper Girdle Imaging in Facioscapulohumeral Muscular Dystrophy
Source: PLoS One. 2014 Jun 16;9(6):e100292. doi: 10.1371/journal.pone.0100292 (PMC4059711; doi:10.1371/journal.pone.0100292)
Supplement: Table S1 — Summary of individual muscle scores across all FSHD patients, progressively ordered by T1-MRI score. (DOCX) [file pone.0100292.s002.docx]

| 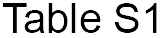1 | 1 | 23 | 33 | 1 | 1 | 0 | 0 | 0 | 0 | 0 | 0 | 0 | 0 | 0 | 0 | 0 | 0 | 0 | 0 | 1 |
| --- | --- | --- | --- | --- | --- | --- | --- | --- | --- | --- | --- | --- | --- | --- | --- | --- | --- | --- | --- | --- |
|  |  |  |  |  | 2 | 0 | 0 | 0 | **1*** | 0 | 0 | 0 | 0 | 0 | 0 | 0 | 0 | 0 | 0 |  |
| 2 | 2 | 38 | 31 | 1 | 1 | 0 | 0 | 0 | 1 | 0 | 0 | 0 | 0 | 0 | 0 | 0 | 0 | 0 | 0 | 1 |
|  |  |  |  |  | 2 | 0 | 0 | 0 | 0 | 0 | 0 | 0 | 0 | 0 | 0 | 0 | 0 | 0 | 0 |  |
| 3 | 1 | 42 | 21 | 1,5 | 1 | 0 | 0 | 0 | 0 | 0 | 0 | 0 | 0 | 0 | 0 | 0 | 0 | 0 | 0 | 2 |
|  |  |  |  |  | 2 | 0 | 0 | 0 | 2 | 0 | 0 | 0 | 0 | 0 | 0 | 0 | 0 | 0 | 0 |  |
| 4 | 2 | 39 | 18 | 2,5 | 1 | 0 | 0 | 0 | 0 | 0 | 0 | 0 | 0 | 0 | 0 | 0 | 0 | 0 | 0 | 2 |
|  |  |  |  |  | 2 | 0 | 0 | 0 | **2*** | 0 | 0 | 0 | 0 | 0 | 0 | 0 | 0 | 0 | 0 |  |
| 5 | 1 | 33 | 27 | 1,5 | 1 | 0 | 0 | 0 | 2 | 0 | 0 | 0 | 0 | 0 | 0 | 0 | 0 | 0 | 0 | 2 |
|  |  |  |  |  | 2 | 0 | 0 | 0 | 0 | 0 | 0 | 0 | 0 | 0 | 0 | 0 | 0 | 0 | 0 |  |
| 6 | 2 | 31 | 15 | 1,5 | 1 | 0 | 0 | 0 | 2 | 0 | 0 | 0 | 0 | 0 | 0 | 0 | 0 | 0 | 0 | 2 |
|  |  |  |  |  | 2 | 0 | 0 | 0 | 0 | 0 | 0 | 0 | 0 | 0 | 0 | 0 | 0 | 0 | 0 |  |
| 7 | 2 | 48 | 23 | 1,5 | 1 | 0 | 0 | 0 | 0 | 0 | 0 | 0 | 0 | 0 | 0 | 0 | 0 | 0 | 0 | 3 |
|  |  |  |  |  | 2 | 0 | 0 | 0 | 3 | 0 | 0 | 0 | 0 | 0 | 0 | 0 | 0 | 0 | 0 |  |
| 8 | 1 | 27 | 27 | 1 | 1 | 0 | 0 | 0 | 1 | 0 | 0 | 0 | 0 | 0 | 0 | 0 | 0 | 0 | 0 | 3 |
|  |  |  |  |  | 2 | 0 | 0 | 0 | 2 | 0 | 0 | 0 | 0 | 0 | 0 | 0 | 0 | 0 | 0 |  |
| 9 | 1 | 19 | 26 | 1,5 | 1 | 0 | 0 | 0 | 2 | 0 | 0 | 0 | 0 | 0 | 0 | 0 | 0 | 0 | 0 | 3 |
|  |  |  |  |  | 2 | 0 | 0 | 0 | 1 | 0 | 0 | 0 | 0 | 0 | 0 | 0 | 0 | 0 | 0 |  |
| 10 | 2 | 31 | 22 | 2,5 | 1 | 0 | 0 | 0 | 0 | 0 | 0 | 1 | 0 | 0 | 0 | 1 | 0 | 0 | 0 | 3 |
|  |  |  |  |  | 2 | 0 | 0 | 0 | 1 | 0 | 0 | 0 | 0 | 0 | 0 | 0 | 0 | 0 | 0 |  |
| 11 | 2 | 23 | 26 | 1 | 1 | 0 | 0 | 0 | 3 | 0 | 0 | 0 | 0 | 0 | 0 | 0 | 0 | 0 | 0 | 4 |
|  |  |  |  |  | 2 | 0 | 0 | 0 | 1 | 0 | 0 | 0 | 0 | 0 | 0 | 0 | 0 | 0 | 0 |  |
| 12 | 2 | 17 | 29 | 1 | 1 | 0 | 0 | 0 | 2 | 0 | 0 | 0 | 0 | 0 | 0 | 0 | 0 | 0 | 0 | 4 |
|  |  |  |  |  | 2 | 0 | 0 | 0 | 2 | 0 | 0 | 0 | 0 | 0 | 0 | 0 | 0 | 0 | 0 |  |
| 13 | 1 | 36 | 30 | 1,5 | 1 | 0 | 0 | 0 | 2 | 0 | 0 | 0 | 0 | 0 | 0 | 0 | 0 | 0 | 0 | 4 |
|  |  |  |  |  | 2 | 0 | 0 | 0 | 2 | 0 | 0 | 0 | 0 | 0 | 0 | 0 | 0 | 0 | 0 |  |
| 14 | 1 | 25 | 26 | 3 | 1 | 0 | 0 | 0 | 3 | 0 | 0 | 0 | 0 | 0 | 0 | 0 | 0 | 0 | 0 | 4 |
|  |  |  |  |  | 2 | 0 | 0 | 0 | 0 | 0 | 0 | 0 | 0 | 0 | 0 | 0 | 0 | 1 | 0 |  |
| 15 | 2 | 17 | 21 | 1,5 | 1 | 0 | 0 | 0 | 1 | 0 | 0 | 1 | 0 | 0 | 0 | 0 | 0 | 0 | 0 | 4 |
|  |  |  |  |  | 2 | 0 | 0 | 0 | 2 | 0 | 0 | 0 | 0 | 0 | 0 | 0 | 0 | 0 | 0 |  |
| 16 | 1 | 26 | 40 | 1,5 | 1 | 0 | 0 | 0 | 2 | 0 | 0 | 1 | 0 | 0 | 0 | 0 | 0 | 0 | 0 | 4 |
|  |  |  |  |  | 2 | 0 | 0 | 0 | 0 | 0 | 0 | 1 | 0 | 0 | 0 | 0 | 0 | 0 | 0 |  |
| 17 | 1 | 39 | 33 | 3 | 1 | 0 | 0 | 0 | 3 | 0 | 0 | 0 | 0 | 0 | 0 | 0 | 0 | 1 | 0 | 5 |
|  |  |  |  |  | 2 | 0 | 0 | 0 | 0 | 0 | 0 | 0 | 0 | 0 | 0 | **0*** | 0 | 1 | 0 |  |
| 18 | 1 | 49 | 32 | 1 | 1 | 0 | 0 | 0 | 0 | 0 | 0 | 1 | 0 | 0 | 0 | 1 | 0 | 0 | 0 | 5 |
|  |  |  |  |  | 2 | 1 | 0 | 0 | 2 | 0 | 0 | 0 | 0 | 0 | 0 | 0 | 0 | 0 | 0 |  |
| 19 | 2 | 40 | 19 | 1 | 1 | 0 | 0 | 0 | 3 | 0 | 0 | 1 | 0 | 0 | 0 | 0 | 0 | 0 | 0 | 6 |
|  |  |  |  |  | 2 | 0 | 0 | 0 | 2 | 0 | 0 | 0 | 0 | 0 | 0 | 0 | 0 | 0 | 0 |  |
|  |  |  |  |  |  |  |  |  |  |  |  |  |  |  |  |  |  |  |  |  |
| 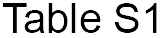20 | 2 | 65 | 21 | 3 | 1 | 0 | 0 | 2 | 1 | 0 | 0 | 0 | 0 | 0 | 0 | 0 | 0 | 0 | 0 | 6 |
|  |  |  |  |  | 2 | 0 | 0 | 2 | 1 | 0 | 0 | 0 | 0 | 0 | 0 | 0 | 0 | 0 | 0 |  |
| 21 | 1 | 22 | 40 | 1,5 | 1 | 0 | 0 | 0 | 3 | 0 | 0 | 1 | 0 | 0 | 0 | 0 | 0 | 0 | 0 | 7 |
|  |  |  |  |  | 2 | 0 | 0 | 0 | 3 | 0 | 0 | **0*** | 0 | 0 | 0 | 0 | 0 | 0 | 0 |  |
| 22 | 1 | 21 | 26 | 1,5 | 1 | 0 | 0 | 0 | 2 | 0 | 0 | 0 | 0 | 0 | 0 | 0 | 0 | 0 | 0 | 7 |
|  |  |  |  |  | 2 | 0 | 0 | 0 | 3 | 0 | 0 | 2 | 0 | 0 | 0 | 0 | 0 | 0 | 0 |  |
| 23 | 1 | 23 | 15 | 3 | 1 | 0 | 0 | 0 | 2 | 0 | 0 | 1 | 0 | 0 | 0 | 0 | 0 | 0 | 0 | 7 |
|  |  |  |  |  | 2 | 0 | 0 | 0 | 2 | 0 | 0 | 2 | 0 | 0 | 0 | 0 | 0 | 0 | 0 |  |
| 24 | 1 | 34 | 30 | 1,5 | 1 | 0 | 0 | 0 | 3 | 0 | 0 | 2 | 0 | 0 | 0 | 1 | 0 | 0 | 0 | 8 |
|  |  |  |  |  | 2 | 0 | 0 | 0 | 2 | 0 | 0 | 0 | 0 | 0 | 0 | 0 | 0 | 0 | 0 |  |
| 25 | 1 | 50 | 23 | 1,5 | 1 | 0 | 1 | 0 | 1 | 0 | 1 | 1 | 0 | 0 | 0 | 0 | 0 | 0 | 0 | 8 |
|  |  |  |  |  | 2 | 0 | 1 | 0 | 2 | 0 | 0 | 1 | 0 | 0 | 0 | 0 | 0 | 0 | 0 |  |
| 26 | 1 | 46 | 35 | 3 | 1 | 0 | 0 | 0 | 3 | 0 | 0 | **2*** | 0 | 0 | 0 | 1 | 0 | **2*** | 0 | 9 |
|  |  |  |  |  | 2 | 0 | 0 | 0 | 0 | 0 | 0 | 0 | 0 | 0 | 0 | 0 | 0 | 1 | 0 |  |
| 27 | 1 | 8 | 13 | 1,5 | 1 | 2 | 0 | 0 | 1 | 0 | 0 | 0 | 0 | 0 | 0 | 0 | 0 | 1 | **1*** | 9 |
|  |  |  |  |  | 2 | 2 | 0 | 0 | 1 | 0 | 0 | 0 | 0 | 0 | 0 | 0 | 0 | 1 | 0 |  |
| 28 | 2 | 41 | 22 | 1,5 | 1 | 0 | 0 | 1 | 2 | 0 | 0 | 1 | 0 | 0 | 0 | 0 | 0 | 1 | 0 | 10 |
|  |  |  |  |  | 2 | 0 | 0 | 1 | 2 | 0 | 0 | 1 | 0 | 0 | 0 | **0*** | 0 | 1 | 0 |  |
| 29 | 2 | 60 | 20 | 3 | 1 | 0 | 0 | 1 | 2 | 0 | 0 | 1 | 0 | 0 | 0 | 0 | 0 | 1 | 0 | 10 |
|  |  |  |  |  | 2 | 0 | 0 | 1 | 2 | 0 | 0 | 1 | 0 | 0 | 0 | 0 | 0 | 1 | 0 |  |
| 30 | 2 | 61 | 27 | 3 | 1 | 0 | 0 | 2 | 2 | 0 | 0 | 1 | 0 | 0 | 0 | 0 | 0 | 1 | 0 | 11 |
|  |  |  |  |  | 2 | 0 | 0 | 2 | 2 | 0 | 0 | 1 | 0 | 0 | 0 | 0 | 0 | 0 | 0 |  |
| 31 | 1 | 42 | 26 | 2,5 | 1 | 0 | 0 | 0 | 3 | 0 | 0 | 2 | 0 | 0 | 0 | **1*** | 0 | 1 | 0 | 11 |
|  |  |  |  |  | 2 | 0 | 0 | 0 | 2 | 0 | 0 | 0 | 0 | 0 | 0 | **1*** | 0 | 1 | 0 |  |
| 32 | 2 | 33 | 21 | 1,5 | 1 | 0 | 0 | 0 | 2 | 0 | 0 | **1*** | 0 | 0 | 0 | **1*** | 1 | 1 | 0 | 11 |
|  |  |  |  |  | 2 | 0 | 0 | 0 | 3 | 0 | 0 | 0 | 0 | 0 | 0 | 1 | 0 | 1 | 0 |  |
| 33 | 1 | 45 | 39 | 3 | 1 | 0 | 0 | 0 | 2 | 0 | 0 | 1 | 0 | 0 | 0 | 2 | 0 | 1 | 0 | 11 |
|  |  |  |  |  | 2 | 0 | 0 | 0 | 2 | 0 | 0 | 1 | 0 | 0 | 0 | 1 | 0 | 1 | 0 |  |
| 34 | 2 | 56 | 32 | 4 | 1 | 0 | 0 | 0 | 1 | 0 | 0 | 2 | 0 | 0 | 0 | 1 | 0 | 1 | 0 | 11 |
|  |  |  |  |  | 2 | 0 | 0 | 0 | 2 | 0 | 0 | 1 | 0 | 0 | 0 | 1 | 1 | 1 | 0 |  |
| 35 | 2 | 30 | 21 | 1 | 1 | 0 | 0 | 1 | 2 | 0 | 0 | 1 | 0 | 0 | 0 | 1 | 0 | 1 | 0 | 11 |
|  |  |  |  |  | 2 | 0 | 0 | 1 | 1 | 0 | 0 | 1 | 0 | 0 | 0 | 1 | 0 | 1 | 0 |  |
| 36 | 2 | 51 | 23 | 3 | 1 | 0 | 0 | 2 | 2 | 0 | 0 | 2 | 0 | 0 | 0 | 0 | 0 | 0 | 0 | 12 |
|  |  |  |  |  | 2 | 0 | 0 | 2 | 2 | 0 | 0 | 2 | 0 | 0 | 0 | 0 | 0 | 0 | 0 |  |
| 37 | 2 | 55 | 23 | 3,5 | 1 | 0 | 0 | 0 | 3 | 0 | 0 | 1 | 0 | 0 | 0 | 1 | 0 | 1 | 0 | 12 |
|  |  |  |  |  | 2 | 0 | 0 | 0 | 3 | 0 | 0 | 1 | 0 | 0 | 0 | 1 | 0 | 1 | 0 |  |
| 38 | 1 | 43 | 23 | 3 | 1 | 0 | 0 | 0 | 3 | 0 | 0 | 1 | 0 | 0 | 0 | 1 | 0 | 1 | 0 | 13 |
|  |  |  |  |  | 2 | 0 | 0 | 0 | 2 | 0 | 0 | 2 | 0 | 0 | 0 | 1 | 0 | 2 | 0 |  |
|  |  |  |  |  |  |  |  |  |  |  |  |  |  |  |  |  |  |  |  |  |
| 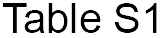39 | 2 | 64 | 31 | 3 | 1 | 0 | 0 | 1 | 3 | 0 | 2 | 1 | 0 | 0 | 0 | 1 | 0 | 0 | 0 | 13 |
|  |  |  |  |  | 2 | 0 | 0 | 1 | 1 | 0 | 0 | 1 | 0 | 0 | 0 | 1 | 0 | 1 | 0 |  |
| 40 | 2 | 63 | 35 | 1 | 1 | 0 | 1 | 2 | 1 | 0 | 0 | 1 | 0 | 0 | 0 | 1 | 0 | 0 | 0 | 13 |
|  |  |  |  |  | 2 | 0 | 1 | 2 | 1 | 0 | 0 | 1 | 0 | 0 | 0 | 1 | 0 | 1 | 0 |  |
| 41 | 1 | 42 | 22 | 4 | 1 | 0 | 0 | 0 | 3 | 0 | 0 | 2 | 0 | 0 | 0 | **2*** | 0 | 2***** | 0 | 14 |
|  |  |  |  |  | 2 | 0 | 0 | 0 | 2 | 0 | 0 | 1 | 0 | 0 | 0 | **1*** | 0 | 1 | 0 |  |
| 42 | 2 | 27 | 22 | 1,5 | 1 | 0 | 0 | 0 | 3 | 0 | 0 | 1 | 0 | 0 | 0 | 1 | 1 | 1 | 0 | 14 |
|  |  |  |  |  | 2 | 0 | 0 | 0 | 3 | 0 | 0 | 1 | 0 | 0 | 0 | 1 | 1 | 1 | 0 |  |
| 43 | 1 | 38 | 26 | 1,5 | 1 | 0 | 0 | 1 | 3 | 0 | 1 | 2 | 0 | 0 | 0 | 1 | 0 | 1 | 0 | 14 |
|  |  |  |  |  | 2 | 0 | 0 | 1 | 2 | 0 | 1 | 0 | 0 | 0 | 0 | 1 | 0 | 0 | 0 |  |
| 44 | 1 | 16 | 23 | 1,5 | 1 | 0 | 0 | 0 | 3 | 0 | 0 | 2 | 0 | 0 | 0 | **2*** | 0 | 3 | 0 | 15 |
|  |  |  |  |  | 2 | 0 | 0 | 0 | 3 | 0 | 0 | **1*** | 0 | 0 | 0 | 1 | 0 | 0 | 0 |  |
| 45 | 2 | 22 | 18 | 3 | 1 | 0 | 0 | 0 | 2 | 0 | 0 | 1 | 0 | 0 | 0 | 1 | 0 | 1 | 1 | 15 |
|  |  |  |  |  | 2 | 0 | 0 | 0 | 0 | 0 | 0 | 2 | 0 | 0 | 0 | 1 | 0 | 3 | 3 |  |
| 46 | 1 | 13 | 18 | 3 | 1 | 2 | 0 | 0 | **2*** | 0 | 0 | 2 | 0 | 0 | 0 | 2 | 0 | 0 | 3 | 16 |
|  |  |  |  |  | 2 | 1 | 0 | 0 | **2*** | 0 | 0 | **1*** | 0 | 0 | 0 | 1 | 0 | 0 | 0 |  |
| 47 | 2 | 31 | 18 | 1,5 | 1 | 0 | 0 | 0 | 3 | 0 | 0 | **1*** | 0 | 0 | 0 | 2 | 0 | 1 | 1 | 16 |
|  |  |  |  |  | 2 | 0 | 0 | 0 | 3 | 0 | 0 | 2 | 0 | 0 | 0 | 1 | 0 | 1 | 1 |  |
| 48 | 2 | 35 | 18 | 2 | 1 | 0 | 0 | 1 | 2 | 0 | 0 | 2 | 0 | 0 | 0 | 2 | 1 | 1 | 0 | 16 |
|  |  |  |  |  | 2 | 0 | 0 | 1 | 2 | 0 | 0 | 2 | 0 | 0 | 0 | **1*** | 0 | 1 | 0 |  |
| 49 | 1 | 50 | 22 | 4 | 1 | 0 | 0 | 1 | 2 | 0 | 0 | 1 | 0 | 0 | 0 | 1 | 0 | 1 | 0 | 16 |
|  |  |  |  |  | 2 | 0 | 0 | 1 | 2 | 0 | 0 | 1 | 0 | 0 | 0 | 2 | 2 | 2 | 0 |  |
| 50 | 1 | 60 | 35 | 3,5 | 1 | 0 | 0 | 2 | 1 | 0 | 0 | 1 | 0 | 0 | 0 | 2 | 0 | 1 | 0 | 16 |
|  |  |  |  |  | 2 | 0 | 0 | 2 | 2 | 0 | 1 | 1 | 0 | 0 | 0 | **1*** | 0 | 2 | 0 |  |
| 51 | 2 | 42 | 31 | 1,5 | 1 | 0 | 0 | 0 | 3 | 0 | 1 | 2 | 0 | 0 | 0 | 1 | 0 | 1 | 1 | 16 |
|  |  |  |  |  | 2 | 0 | 0 | 0 | 2 | 0 | 1 | 1 | 0 | 0 | 0 | 1 | 0 | 1 | 1 |  |
| 52 | 2 | 22 | 22 | 1,5 | 1 | 0 | 0 | 1 | 3 | 0 | 0 | 1 | 0 | 0 | 0 | 1 | 1 | 1 | 0 | 17 |
|  |  |  |  |  | 2 | 0 | 0 | 1 | 2 | 0 | 1 | 2 | 0 | 0 | 0 | 1 | 1 | 1 | 0 |  |
| 53 | 1 | 34 | 21 | 3 | 1 | 0 | 0 | 0 | 3 | 0 | 0 | 2 | 0 | 0 | 0 | 1 | 0 | 3 | 0 | 18 |
|  |  |  |  |  | 2 | 0 | 0 | 0 | 3 | 0 | 0 | 2 | 0 | 0 | 0 | 1 | 0 | 3 | 0 |  |
| 54 | 1 | 51 | 26 | 3 | 1 | 0 | 0 | 2 | 3 | 0 | 0 | 1 | 0 | 0 | 0 | 1 | 0 | 1 | 0 | 19 |
|  |  |  |  |  | 2 | 0 | 0 | 2 | 3 | 0 | 0 | 1 | 0 | 0 | 0 | 3 | 1 | 1 | 0 |  |
| 55 | 1 | 37 | 35 | 1,5 | 1 | 0 | 0 | 0 | 3 | 0 | 1 | 2 | 0 | 0 | 0 | 2 | 1 | 2 | 1 | 19 |
|  |  |  |  |  | 2 | 0 | 0 | 0 | 2 | 0 | 0 | 1 | 0 | 0 | 0 | 1 | 0 | 2 | 1 |  |
| 56 | 2 | 51 | 29 | 2,5 | 1 | 0 | 0 | 2 | 3 | 0 | 0 | 2 | 0 | 0 | 0 | 1 | 0 | 1 | 1 | 19 |
|  |  |  |  |  | 2 | 0 | 0 | 2 | 3 | 0 | 0 | 1 | 0 | 0 | 0 | 1 | 0 | 1 | 1 |  |
| 57 | 2 | 64 | 23 | 3 | 1 | 0 | 2 | 2 | 1 | 0 | 0 | 1 | 0 | 0 | 0 | 1 | 1 | 1 | 0 | 19 |
|  |  |  |  |  | 2 | 0 | 2 | 2 | 2 | 0 | 0 | 1 | 0 | 0 | 0 | 1 | 1 | 1 | 0 |  |
|  |  |  |  |  |  |  |  |  |  |  |  |  |  |  |  |  |  |  |  |  |
| 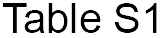58 | 2 | 60 | 26 | 1,5 | 1 | 0 | 1 | 2 | 2 | 0 | 0 | 2 | 0 | 0 | 0 | 1 | 1 | 1 | 0 | 19 |
|  |  |  |  |  | 2 | 0 | 1 | 2 | 1 | 0 | 1 | 1 | 0 | 0 | 0 | 1 | 1 | 1 | 0 |  |
| 59 | 2 | 28 | 26 | 3,5 | 1 | 0 | 0 | 0 | 3 | 0 | 0 | 2 | 0 | 0 | 0 | 1 | 1 | 1 | 3 | 20 |
|  |  |  |  |  | 2 | 0 | 0 | 0 | 3 | 0 | 0 | 1 | 0 | 0 | 0 | 2 | 1 | 1 | 1 |  |
| 60 | 2 | 45 | 30 | 2,5 | 1 | 0 | 0 | 2 | 2 | 0 | 1 | 1 | 0 | 0 | 0 | 1 | 1 | 1 | 1 | 21 |
|  |  |  |  |  | 2 | 0 | 0 | 2 | 3 | 0 | 1 | 1 | 0 | 0 | 0 | 1 | 1 | 1 | 1 |  |
| 61 | 2 | 61 | 27 | 1,5 | 1 | 0 | 2 | 3 | 3 | 0 | 0 | 1 | 0 | 0 | 0 | 1 | 0 | 1 | 0 | 22 |
|  |  |  |  |  | 2 | 0 | 2 | 3 | 2 | 0 | 1 | 1 | 0 | 0 | 0 | 1 | 0 | 1 | 0 |  |
| 62 | 2 | 65 | 26 | 3 | 1 | 0 | 0 | 2 | 2 | 0 | 0 | 1 | 0 | 0 | 0 | 2 | 1 | 2 | 2 | 22 |
|  |  |  |  |  | 2 | 0 | 0 | 2 | 2 | 0 | 0 | 1 | 0 | 0 | 0 | 1 | 1 | 2 | 1 |  |
| 63 | 2 | 67 | 29 | 3 | 1 | 0 | 0 | 2 | 3 | 0 | 2 | 2 | 0 | 0 | 0 | 2 | 1 | 1 | 0 | 22 |
|  |  |  |  |  | 2 | 0 | 0 | 2 | 2 | 0 | 1 | 1 | 0 | 0 | 0 | 1 | 1 | 1 | 0 |  |
| 64 | 1 | 39 | 23 | 2,5 | 1 | 0 | 0 | 0 | 3 | 0 | 3 | 2 | 0 | 0 | 0 | 2 | 2 | 3 | 2 | 24 |
|  |  |  |  |  | 2 | 0 | 0 | 0 | 3 | 0 | 0 | 1 | 0 | 0 | 0 | 2 | 0 | 1 | 0 |  |
| 65 | 2 | 18 | 13 | 3,5 | 1 | 0 | 0 | 0 | 2 | 3 | 3 | 2 | 0 | 0 | 0 | 1 | 0 | 1 | 0 | 24 |
|  |  |  |  |  | 2 | 0 | 0 | 0 | 2 | 3 | 3 | 2 | 0 | 0 | 0 | **1*** | 0 | 1 | 0 |  |
| 66 | 1 | 37 | 31 | 1,5 | 1 | 0 | 0 | 1 | 3 | 0 | 2 | 2 | 0 | 0 | 0 | 2 | 0 | 2 | 0 | 24 |
|  |  |  |  |  | 2 | 2 | 0 | 1 | 3 | 0 | 2 | 1 | 0 | 0 | 0 | **1*** | 0 | 2 | 0 |  |
| 67 | 1 | 53 | 32 | 3,5 | 1 | 0 | 1 | 0 | 3 | 0 | 1 | 2 | 0 | 0 | 0 | 3 | 2 | 2 | 1 | 24 |
|  |  |  |  |  | 2 | 0 | 1 | 0 | 2 | 0 | 0 | 1 | 0 | 0 | 0 | 2 | 0 | 2 | 1 |  |
| 68 | 1 | 45 | 38 | 3,5 | 1 | 0 | 0 | 0 | 3 | 0 | 3 | 2 | 0 | 0 | 0 | 3 | 0 | 3 | 0 | 25 |
|  |  |  |  |  | 2 | 0 | 0 | 0 | 3 | 0 | 1 | 2 | 0 | 0 | 0 | 2 | 0 | 3 | 0 |  |
| 69 | 1 | 45 | 24 | 3,5 | 1 | 0 | 0 | 0 | 3 | 0 | 0 | 2 | 0 | 0 | 0 | 2 | 2 | 3 | 1 | 25 |
|  |  |  |  |  | 2 | 0 | 0 | 0 | 3 | 0 | 0 | 1 | 0 | 0 | 0 | **2*** | 2 | 1 | 3 |  |
| 70 | 2 | 57 | 25 | 4 | 1 | 0 | 0 | 1 | 3 | 3 | 3 | 2 | 0 | 0 | 0 | 1 | 0 | 1 | 0 | 25 |
|  |  |  |  |  | 2 | 0 | 0 | 1 | 3 | 0 | 1 | 2 | 0 | 0 | 0 | **2*** | 1 | 1 | 0 |  |
| 71 | 2 | 42 | 23 | 4 | 1 | 0 | 0 | 0 | 3 | 3 | 3 | 3 | 0 | 0 | 0 | **2*** | 0 | 2 | 0 | 26 |
|  |  |  |  |  | 2 | 0 | 0 | 0 | 3 | 0 | **2*** | 3 | 0 | 0 | 0 | **1*** | 0 | 1 | 0 |  |
| 72 | 1 | 51 | 24 | 3 | 1 | 0 | 0 | 0 | 2 | 0 | **2*** | 2 | 0 | 0 | 0 | 2 | 3 | 2 | 0 | 26 |
|  |  |  |  |  | 2 | 0 | 0 | 0 | 2 | 0 | 2 | 2 | 0 | 0 | 0 | **2*** | 3 | 2 | 0 |  |
| 73 | 2 | 45 | 21 | 4,5 | 1 | 0 | 0 | 0 | 3 | **0*** | 3 | 2 | 0 | 0 | 0 | 2 | 0 | 2 | 1 | 26 |
|  |  |  |  |  | 2 | 0 | 0 | 0 | 3 | **0*** | 3 | **2*** | 0 | 0 | 0 | **2*** | **0*** | **2*** | 1 |  |
| 74 | 2 | 38 | 27 | 4 | 1 | 3 | 0 | 2 | 3 | 0 | 3 | 2 | 0 | 0 | 0 | 2 | 1 | 3 | 0 | 27 |
|  |  |  |  |  | 2 | 0 | 0 | 0 | 2 | 0 | 0 | 2 | 0 | 0 | 0 | 2 | 0 | 2 | 0 |  |
| 75 | 1 | 39 | 28 | 4 | 1 | 1 | 0 | 0 | 3 | 0 | 0 | 2 | 0 | 0 | 0 | 2 | 3 | 2 | 1 | 27 |
|  |  |  |  |  | 2 | 0 | 0 | 0 | 3 | 0 | 0 | 2 | 0 | 0 | 0 | 2 | 3 | 2 | 1 |  |
| 76 | 1 | 49 | 26 | 3 | 1 | 0 | 0 | 0 | 3 | 0 | 1 | 2 | 0 | 0 | 0 | 3 | 0 | 3 | 3 | 28 |
|  |  |  |  |  | 2 | 1 | 0 | 0 | 3 | 0 | 2 | 2 | 0 | 0 | 0 | 2 | 0 | 3 | 0 |  |
|  |  |  |  |  |  |  |  |  |  |  |  |  |  |  |  |  |  |  |  |  |
| 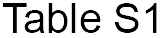77 | 1 | 42 | 30 | 3,5 | 1 | 0 | 0 | 0 | 3 | 3 | 3 | 3 | 0 | 0 | 0 | 2 | 1 | 1 | 0 | 28 |
|  |  |  |  |  | 2 | 0 | 0 | 0 | 3 | 0 | 0 | 2 | 0 | 0 | 0 | 2 | 1 | 1 | 3 |  |
| 78 | 2 | 54 | 26 | 4 | 1 | 0 | 0 | 1 | 3 | 0 | 0 | 3 | 0 | 0 | 0 | 2 | 2 | 2 | 2 | 29 |
|  |  |  |  |  | 2 | 0 | 0 | 1 | 3 | 0 | 0 | 3 | 0 | 0 | 0 | 2 | 1 | 2 | 2 |  |
| 79 | 1 | 63 | 21 | 3,5 | 1 | 0 | 0 | 2 | 3 | 0 | 2 | 2 | 0 | 0 | 0 | 2 | 1 | 3 | 1 | 29 |
|  |  |  |  |  | 2 | 0 | 0 | 2 | 2 | 0 | 2 | 2 | 0 | 0 | 0 | 1 | 0 | 3 | 1 |  |
| 80 | 1 | 38 | 26 | 3 | 1 | 0 | 0 | 0 | 3 | 0 | 1 | 2 | 0 | 0 | 0 | **1*** | 1 | 2 | 2 | 29 |
|  |  |  |  |  | 2 | 3 | 0 | 0 | 3 | 0 | 1 | 2 | 0 | 0 | 0 | **2*** | 1 | 2 | 3 |  |
| 81 | 1 | 45 | 27 | 3 | 1 | 0 | 0 | 0 | 3 | 0 | 3 | 2 | 0 | 0 | 0 | 3 | 3 | 3 | 0 | 30 |
|  |  |  |  |  | 2 | 0 | 0 | 0 | 3 | 0 | 1 | 2 | 0 | 0 | 0 | 1 | 3 | 3 | 0 |  |
| 82 | 2 | 51 | 25 | 4 | 1 | 0 | 0 | 1 | 3 | 0 | 3 | 2 | 0 | 0 | 0 | 2 | 3 | 2 | 1 | 30 |
|  |  |  |  |  | 2 | 0 | 0 | 1 | 3 | 0 | 0 | 2 | 0 | 0 | 0 | 2 | 3 | 2 | 0 |  |
| 83 | 1 | 25 | 23 | 3 | 1 | 3 | 0 | 0 | 3 | 3 | 3 | **2*** | 0 | 0 | 0 | 2 | 0 | 1 | 0 | 30 |
|  |  |  |  |  | 2 | 2 | 0 | 0 | 3 | 0 | 1 | 1 | 0 | 0 | 0 | 2 | 0 | 1 | **3*** |  |
| 84 | 2 | 62 | 35 | 4 | 1 | 0 | 0 | 1 | 2 | 2 | 3 | 3 | 0 | 0 | 0 | 2 | 3 | 3 | 0 | 34 |
|  |  |  |  |  | 2 | 0 | 0 | 1 | 3 | 2 | 2 | 2 | 0 | 0 | 0 | 1 | 2 | 2 | 0 |  |
| 85 | 2 | 42 | 10 | 4,5 | 1 | 2 | 0 | 0 | 2 | 0 | 2 | 3 | 0 | 0 | **0*** | 2 | 2 | 2 | 3 | 34 |
|  |  |  |  |  | 2 | 1 | 0 | 0 | 1 | 0 | 1 | 1 | 3 | 0 | 0 | **2*** | 2 | 2 | 3 |  |
| 86 | 1 | 25 | 26 | 3,5 | 1 | 0 | 0 | 0 | 3 | 2 | 3 | 2 | 0 | 0 | 0 | 2 | 2 | 3 | 1 | 35 |
|  |  |  |  |  | 2 | 0 | 0 | 0 | 3 | 2 | 3 | 2 | 0 | 0 | 0 | 2 | 2 | 3 | 0 |  |
| 87 | 2 | 40 | 19 | 3,5 | 1 | 2 | 0 | 0 | 3 | 0 | 2 | 2 | 0 | 0 | 0 | 3 | 2 | 3 | 0 | 35 |
|  |  |  |  |  | 2 | 2 | 0 | 0 | 3 | 1 | 2 | 2 | 0 | 0 | 0 | 3 | 1 | 3 | 1 |  |
| 88 | 2 | 70 | 17 | 3,5 | 1 | 0 | 2 | 2 | 3 | 0 | 1 | 3 | 0 | 0 | 0 | 2 | 3 | 2 | 1 | 37 |
|  |  |  |  |  | 2 | 0 | 2 | 2 | 3 | 0 | 1 | 3 | 0 | 0 | 0 | 1 | 3 | 2 | 1 |  |
| 89 | 1 | 57 | 29 | 3,5 | 1 | 1 | 1 | 2 | 3 | 0 | 3 | 3 | 0 | 0 | 0 | 2 | 1 | 3 | 0 | 38 |
|  |  |  |  |  | 2 | 1 | 1 | 2 | 3 | 0 | 3 | 3 | 0 | 0 | 0 | 2 | 1 | 2 | 1 |  |
| 90 | 2 | 23 | 17 | 4 | 1 | 3 | 0 | 0 | 3 | 0 | 2 | 2 | 0 | 0 | 0 | 3 | 3 | 3 | 0 | 39 |
|  |  |  |  |  | 2 | 3 | 0 | 0 | 3 | 0 | 2 | 3 | 0 | 0 | 0 | 3 | 3 | 3 | 0 |  |
| 91 | 2 | 42 | 17 | 4 | 1 | 0 | 0 | 0 | 3 | 3 | 3 | 3 | 0 | 0 | 0 | 2 | 3 | 2 | 1 | 39 |
|  |  |  |  |  | 2 | 2 | 0 | 0 | **2*** | 3 | 3 | 3 | 0 | 0 | 0 | 2 | 1 | 2 | 1 |  |
| 92 | 2 | 68 | 40 | 4,5 | 1 | 0 | 1 | 2 | 3 | 1 | 1 | 2 | 0 | 0 | 0 | 2 | 2 | 2 | 3 | 39 |
|  |  |  |  |  | 2 | 0 | 1 | 2 | 3 | 1 | 3 | 2 | 0 | 0 | 0 | 2 | 1 | 2 | 3 |  |
| 93 | 1 | 39 | 23 | 3,5 | 1 | 3 | 0 | 0 | 3 | 0 | 3 | 3 | 0 | 0 | 0 | 3 | 3 | 3 | 0 | 40 |
|  |  |  |  |  | 2 | 3 | 0 | 0 | 3 | 0 | 3 | 3 | 0 | 0 | 0 | 3 | 1 | 3 | 0 |  |
| 94 | 1 | 62 | 27 | 3,5 | 1 | 1 | 0 | 2 | 3 | 0 | 2 | 2 | 0 | 0 | 1 | 2 | 1 | 3 | 3 | 40 |
|  |  |  |  |  | 2 | 0 | 0 | 2 | 3 | **2*** | 3 | 2 | 0 | 0 | 1 | 2 | 1 | 3 | 1 |  |
| 95 | 2 | 43 | 15 | 4 | 1 | 1 | 1 | 2 | 3 | 1 | **1*** | 2 | 1 | 1 | 0 | 2 | 2 | 2 | 1 | 40 |
|  |  |  |  |  | 2 | 1 | 1 | 2 | 3 | **1*** | **2*** | 2 | 1 | 1 | 0 | 2 | 2 | 1 | 1 |  |
|  |  |  |  |  |  |  |  |  |  |  |  |  |  |  |  |  |  |  |  |  |
| 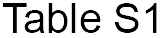96 | 1 | 42 | 19 | 3,5 | 1 | 2 | 0 | 2 | 3 | 0 | 3 | 3 | 0 | 0 | 0 | 3 | 3 | 3 | 1 | 41 |
|  |  |  |  |  | 2 | 0 | 0 | 2 | 3 | 0 | 1 | 2 | 0 | 0 | 0 | 3 | 3 | 3 | 1 |  |
| 97 | 2 | 42 | 22 | 4 | 1 | 2 | 0 | 0 | 3 | 3 | 3 | 3 | 0 | 0 | 0 | 2 | 3 | 3 | 1 | 41 |
|  |  |  |  |  | 2 | 2 | 0 | 0 | 3 | 2 | 2 | 3 | 0 | 0 | 0 | 2 | 2 | 1 | 1 |  |
| 98 | 2 | 45 | 20 | 4 | 1 | 0 | 2 | 3 | 3 | 0 | 1 | 1 | 0 | 0 | 0 | 2 | 2 | 3 | 2 | 42 |
|  |  |  |  |  | 2 | 0 | 2 | 3 | 3 | 2 | 3 | **1*** | 0 | 0 | 0 | 3 | 1 | 3 | 2 |  |
| 99 | 2 | 38 | 15 | 4,5 | 1 | 3 | 0 | 0 | 3 | 3 | 3 | 3 | 0 | 0 | 0 | 3 | 2 | 3 | 0 | 43 |
|  |  |  |  |  | 2 | 3 | 0 | 0 | 3 | 0 | 2 | 2 | 0 | 0 | 0 | 2 | 2 | 3 | 3 |  |
| 100 | 2 | 34 | 19 | 4,5 | 1 | 3 | 0 | 0 | 3 | 0 | 3 | 3 | 0 | 0 | 0 | 3 | 3 | 3 | 0 | 44 |
|  |  |  |  |  | 2 | 3 | 0 | 0 | 3 | 0 | 3 | 2 | 0 | 0 | 0 | 3 | 3 | 3 | 3 |  |
| 101 | 1 | 46 | 40 | 4 | 1 | 1 | 0 | 0 | 3 | 3 | 3 | 3 | 0 | 0 | 0 | 2 | 3 | 3 | 2 | 44 |
|  |  |  |  |  | 2 | 1 | 0 | 0 | 3 | 0 | 3 | 2 | 0 | 0 | 0 | 3 | 3 | 3 | 3 |  |
| 102 | 2 | 65 | 23 | 3 | 1 | 0 | 2 | 3 | 3 | 0 | 1 | 3 | 1 | 1 | **1*** | 2 | 2 | 3 | 1 | 45 |
|  |  |  |  |  | 2 | 0 | 2 | 3 | 2 | 0 | 3 | 3 | 0 | 0 | 0 | 3 | 2 | 3 | 1 |  |
| 103 | 1 | 61 | 26 | 4 | 1 | 2 | 0 | 1 | 3 | 2 | 3 | 3 | 0 | 0 | 0 | 2 | 3 | 3 | 1 | 46 |
|  |  |  |  |  | 2 | 2 | 0 | 1 | 3 | 2 | 3 | 3 | 0 | 0 | 0 | 3 | 1 | 3 | 2 |  |
| 104 | 2 | 18 | 18 | 4 | 1 | 3 | 0 | 0 | 3 | 3 | 3 | 3 | 0 | 0 | 0 | 3 | 3 | 3 | 0 | 47 |
|  |  |  |  |  | 2 | 3 | 0 | 0 | 3 | 0 | 2 | **3*** | 0 | 0 | 0 | 3 | 3 | 3 | 3 |  |
| 105 | 1 | 34 | 20 | 4 | 1 | 1 | 0 | 2 | 3 | 2 | 3 | 3 | 0 | **2*** | 0 | 3 | 3 | 3 | 3 | 51 |
|  |  |  |  |  | 2 | 2 | 0 | 2 | 3 | 1 | 3 | 2 | 0 | 1 | 0 | 3 | 0 | 3 | 3 |  |
| 106 | 1 | 11 | 13 | 4,5 | 1 | 3 | 0 | 1 | 3 | 3 | 3 | 2 | 0 | **0*** | 0 | 3 | 3 | 3 | 3 | 52 |
|  |  |  |  |  | 2 | 3 | 0 | 1 | 3 | 2 | **2*** | 2 | 0 | 0 | 0 | 3 | 3 | 3 | 3 |  |
| 107 | 2 | 68 | 21 | 3,5 | 1 | 0 | 2 | 3 | 3 | 3 | 3 | 3 | 0 | 0 | 0 | 3 | 3 | 3 | 1 | 52 |
|  |  |  |  |  | 2 | 1 | 2 | 3 | 3 | 3 | 3 | 2 | 0 | 0 | 0 | 3 | 2 | 3 | 0 |  |
| 108 | 1 | 41 | 21 | 4,5 | 1 | 0 | 2 | 1 | 3 | 3 | 3 | 3 | 1 | 1 | 1 | 3 | 3 | 3 | 3 | 59 |
|  |  |  |  |  | 2 | 0 | 2 | 1 | 3 | 3 | 3 | 2 | 1 | 1 | 1 | 3 | 3 | 3 | 3 |  |

*****: hyperintense on STIR sequences

## Table S1

Summary of individual muscle scores across all FSHD patients, progressively ordered by T1-MRI score
