# Supplementary material for: Upper Girdle Imaging in Facioscapulohumeral Muscular Dystrophy
Source: PLoS One. 2014 Jun 16;9(6):e100292. doi: 10.1371/journal.pone.0100292 (PMC4059711; doi:10.1371/journal.pone.0100292)
Supplement: Table S2 — Summary of individual muscle scores across all the non-FSHD patients, subdivided by diagnosis. In LGMD2L asymmetric involvement is a feature, but the teres major is the most involved muscle, followed by thoracic paraspinal muscles with relative sparing of trapezius and serratus anterior. In LGMD1D, the serratus anterior is always mildly involved, sometimes together with spinati or subscapularis, and the trapezius is always spared. In manifesting female carriers of dystrophinopathy, asymmetrical involvement is present together with trapezius and serratus anterior involvement, but heavy and early involvement of supra- and infraspinatus, pectoralis minor and subscapularis help in the differential diagnosis with FSHD. In HMERF, the sternocleidomastoid is always affected and the trapezius is always spared. In SLONM, serratus anterior and subscapularis are more affected than trapezius. In MYH7-related myopathy, the sternocleidomastoid is heavily affected in the more severe patient and subscapularis involvement would make the diagnosis of FSHD unlikely in the milder one. In TPM2-related myopathy, the trapezius is always spared and thoracic paraspinal and neck extensors are affected. STIR hyperintensities could be found only in a minority of non-FSHD patients. Differences between LGMD2A, LGMD2B, AMD and FSHD patterns are discussed in the text. (DOCX) [file pone.0100292.s003.docx]

**LGMD2A**

| **Patient ID** | **Sex (M=1;F=2)** | **Age (years)** | **Side (right=1;left=2)** | **Sternocleidomastoid** | **Neck extensors** | **Thoracic paraspinal** | **Trapezius** | **Levator scapulae** | **Rhomboids** | **Serratus anterior** | **Supraspinatus** | **Infraspinatus** | **Subscapularis** | **Pectoralis major** | **Pectoralis minor** | **Latissimus dorsi** | **Teres major** | **T1-MRI score** |
| --- | --- | --- | --- | --- | --- | --- | --- | --- | --- | --- | --- | --- | --- | --- | --- | --- | --- | --- |
| 1 | 2 | 24 | 1 | 0 | 1 | 1 | 2 | 2 | 1 | 3 | 2 | 1 | 2 | 2 | 3 | 1 | 1 | 43 |
|  |  |  | 2 | 0 | 1 | 1 | 2 | 2 | 1 | 2 | 1 | 1 | 2 | 2 | 3 | 2 | 1 |  |
| 2 | 1 | 48 | 1 | 0 | 2 | 2 | 3 | 1 | 3 | 3 | 2 | 2 | 3 | 3 | 3 | 3 | 3 | 65 |
|  |  |  | 2 | 0 | 2 | 2 | 3 | 2 | 3 | 3 | 1 | 1 | 3 | 3 | 3 | 3 | 3 |  |
| 3 | 2 | 43 | 1 | 3 | 1 | 1 | 3 | 3 | 3 | 3 | 3 | 3 | 3 | 3 | 3 | 3 | 3 | 74 |
|  |  |  | 2 | 1 | 1 | 1 | 3 | 3 | 3 | 3 | 3 | 3 | 3 | 3 | 3 | 3 | 3 |  |
| 4 | 2 | 68 | 1 | 0 | 2 | 3 | 3 | 3 | 3 | 3 | 3 | 3 | 3 | 3 | 3 | 3 | 3 | 76 |
|  |  |  | 2 | 0 | 2 | 3 | 3 | 3 | 3 | 3 | 3 | 3 | 3 | 3 | 3 | 3 | 3 |  |
| 5 | 1 | 37 | 1 | 3 | 2 | 2 | 3 | 3 | 3 | 3 | 3 | 3 | 3 | 3 | 3 | 3 | 3 | 80 |
|  |  |  | 2 | 3 | 2 | 2 | 3 | 3 | 3 | 3 | 3 | 3 | 3 | 3 | 3 | 3 | 3 |  |
| 6 | 2 | 37 | 1 | 0 | 1 | 1 | 2 | 1 | 1 | 3 | 2 | 1 | 3 | 2 | 2 | 2 | 3 | 48 |
|  |  |  | 2 | 0 | 1 | 1 | 2 | 1 | 1 | 3 | 2 | 1 | 3 | 2 | 2 | 2 | 3 |  |
| 7 | 2 | 39 | 1 | 2 | 1 | 2 | 2 | 2 | 3 | 3 | 2 | 1 | 3 | 2 | 2 | 3 | 3 | 60 |
|  |  |  | 2 | 2 | 1 | 2 | 2 | 2 | 2 | 3 | 2 | 1 | 3 | 2 | 2 | 2 | 3 |  |
| 8 | 1 | 37 | 1 | 0 | 1 | 1 | 2 | 0 | 1 | 2 | 1 | 0 | 1 | 2 | 2 | 2 | 3 | 35 |
|  |  |  | 2 | 0 | 1 | 1 | 2 | 0 | 1 | 2 | 1 | 0 | 1 | 2 | 2 | 2 | 2 |  |
| 9 | 2 | 53 | 1 | 0 | 1 | 1 | 2 | 2 | 2 | 3 | **2*** | 1 | 3 | 2 | 2 | 3 | 3 | 56 |
|  |  |  | 2 | 0 | 1 | 1 | 2 | 2 | 2 | 3 | 3 | 2 | 3 | 2 | 2 | 3 | 3 |  |
| 10 | 2 | 42 | 1 | 1 | 1 | 2 | 2 | 1 | 3 | 3 | 2 | 2 | 2 | 2 | 2 | 2 | 3 | 53 |
|  |  |  | 2 | 1 | 1 | 2 | 2 | 1 | 1 | 3 | 1 | 2 | 2 | 2 | 2 | 2 | 3 |  |
| 11 | 2 | 68 | 1 | 0 | 1 | 2 | 3 | 0 | 1 | 2 | 0 | 0 | 1 | 2 | 3 | 3 | 0 | 35 |
|  |  |  | 2 | 0 | 1 | 2 | 3 | 0 | 1 | 1 | 0 | 0 | 2 | 2 | 3 | 2 | 0 |  |
| 12 | 2 | 39 | 1 | 2 | 1 | 2 | 3 | 3 | 3 | 3 | 3 | **3*** | 3 | 3 | 3 | 3 | 3 | 76 |
|  |  |  | 2 | 2 | 1 | 2 | 3 | 3 | 3 | 3 | 3 | 3 | 3 | 3 | 3 | 3 | 3 |  |
| 13 | 1 | 56 | 1 | 0 | 1 | 1 | 2 | 1 | 2 | 3 | 2 | 1 | 3 | 3 | 3 | 3 | 3 | 56 |
|  |  |  | 2 | 0 | 1 | 1 | 2 | 1 | 2 | 3 | 2 | 1 | 3 | 3 | 3 | 3 | 3 |  |
| 14 | 2 | 50 | 1 | 0 | 0 | 2 | 2 | 2 | 3 | 3 | 0 | 0 | 3 | 2 | 3 | 3 | 2 | 50 |
|  |  |  | 2 | 0 | 0 | 2 | 2 | 2 | 3 | 3 | 0 | 0 | 3 | 2 | 3 | 3 | 2 |  |

*****: hyperintense on STIR sequences

| **Patient ID** | **Sex (M=1;F=2)** | **Age (years)** | **Side (right=1;left=2)** | **Sternocleidomastoid** | **Neck extensors** | **Thoracic paraspinal** | **Trapezius** | **Levator scapulae** | **Rhomboids** | **Serratus anterior** | **Supraspinatus** | **Infraspinatus** | **Subscapularis** | **Pectoralis major** | **Pectoralis minor** | **Latissimus dorsi** | **Teres major** | **T1-MRI score** |
| --- | --- | --- | --- | --- | --- | --- | --- | --- | --- | --- | --- | --- | --- | --- | --- | --- | --- | --- |
| 1 | 1 | 45 | 1 | 1 | 1 | 2 | **2*** | 0 | 1 | 1 | 1 | 1 | 2 | 2 | 1 | 2 | 2 | 40 |
|  |  |  | 2 | 1 | 1 | 2 | 1 | 2 | 1 | 1 | 2 | 1 | 2 | 1 | 1 | 2 | 3 |  |
| 2 | 2 | 50 | 1 | 1 | 1 | 2 | 1 | 0 | 1 | 2 | 3 | 2 | 3 | 2 | 1 | 3 | 3 | 48 |
|  |  |  | 2 | 1 | 1 | 2 | 1 | 0 | 1 | 1 | 3 | 2 | 3 | 3 | 0 | 2 | 3 |  |
| 3 | 2 | 39 | 1 | 1 | 1 | 1 | 2 | 0 | 2 | 0 | 2 | 1 | 2 | 2 | 1 | 2 | 3 | 39 |
|  |  |  | 2 | 1 | 1 | 1 | 1 | 0 | 1 | 0 | 2 | 1 | 2 | 3 | 1 | 2 | 3 |  |
| 4 | 2 | 48 | 1 | 0 | 1 | 1 | 1 | 0 | 1 | 1 | 2 | 1 | 3 | 2 | 1 | 1 | 3 | 36 |
|  |  |  | 2 | 0 | 1 | 1 | 1 | 0 | 1 | 1 | 2 | 1 | 3 | 2 | 1 | 1 | 3 |  |
| 5 | 1 | 31 | 1 | 1 | 1 | 0 | 1 | 0 | 0 | 0 | 2 | 1 | 2 | **1*** | 0 | 1 | 2 | 24 |
|  |  |  | 2 | 1 | 1 | 0 | 1 | 0 | 0 | 0 | 2 | 1 | 2 | **1*** | 0 | 1 | 2 |  |

**LGMD2B/MM**

*****: hyperintense on STIR sequences

**LGMD2L**

| **Patient ID** | **Sex (M=1;F=2)** | **Age (years)** | **Side (right=1;left=2)** | **Sternocleidomastoid** | **Neck extensors** | **Thoracic paraspinal** | **Trapezius** | **Levator scapulae** | **Rhomboids** | **Serratus anterior** | **Supraspinatus** | **Infraspinatus** | **Subscapularis** | **Pectoralis major** | **Pectoralis minor** | **Latissimus dorsi** | **Teres major** | **T1-MRI score** |
| --- | --- | --- | --- | --- | --- | --- | --- | --- | --- | --- | --- | --- | --- | --- | --- | --- | --- | --- |
| 1 | 1 | 64 | 1 | 0 | 0 | 2 | 1 | 0 | 0 | 1 | 2 | 2 | 0 | 2 | 0 | 0 | 3 | 21 |
|  |  |  | 2 | 0 | 0 | 2 | 1 | 0 | 0 | 1 | 0 | 0 | 0 | 1 | 0 | 0 | 3 |  |
| 2 | 1 | 65 | 1 | 0 | 0 | 2 | 0 | 0 | 0 | 1 | 0 | 0 | 0 | 0 | 0 | 1 | 3 | 11 |
|  |  |  | 2 | 0 | 0 | 2 | 0 | 0 | 0 | 1 | 0 | 0 | 0 | 0 | 0 | 1 | 0 |  |
| 3 | 1 | 44 | 1 | 0 | 0 | 2 | 0 | 0 | 0 | 1 | 0 | 0 | 0 | 1 | 0 | 1 | 3 | 16 |
|  |  |  | 2 | 0 | 0 | 2 | 0 | 0 | 0 | 1 | 0 | 0 | 0 | 1 | 0 | 1 | 3 |  |

**Acid Maltase Deficiency**

| **Patient ID** | **Sex (M=1;F=2)** | **Age (years)** | **Side (right=1;left=2)** | **Sternocleidomastoid** | **Neck extensors** | **Thoracic paraspinal** | **Trapezius** | **Levator scapulae** | **Rhomboids** | **Serratus anterior** | **Supraspinatus** | **Infraspinatus** | **Subscapularis** | **Pectoralis major** | **Pectoralis minor** | **Latissimus dorsi** | **Teres major** | **T1-MRI score** |
| --- | --- | --- | --- | --- | --- | --- | --- | --- | --- | --- | --- | --- | --- | --- | --- | --- | --- | --- |
| 1 | 2 | 44 | 1 | 0 | 0 | 0 | 0 | 0 | 0 | 0 | 0 | 0 | 0 | 0 | 0 | 0 | 0 | 1 |
|  |  |  | 2 | 0 | 0 | 0 | 1 | 0 | 0 | 0 | 0 | 0 | 0 | 0 | 0 | 0 | 0 |  |
| 2 | 1 | 39 | 1 | 0 | 0 | 0 | 2 | 3 | 3 | 3 | 0 | 1 | 3 | 3 | 3 | 3 | 3 | 51 |
|  |  |  | 2 | 0 | 0 | 0 | **0*** | 3 | 2 | 3 | 0 | 1 | 3 | 3 | 3 | 3 | 3 |  |
| 3*^†^* | 1 | 48 | 1 | 0 | 0 | 1 | 1 | 0 | 0 | 1 | 0 | 0 | 2 | 2 | 3 | 3 | 0 | 27 |
|  |  |  | 2 | 0 | 0 | 1 | 2 | 0 | 0 | 1 | 0 | 0 | 2 | 2 | 3 | 3 | 0 |  |
| 4 | 2 | 63 | 1 | 0 | 0 | 2 | 0 | 0 | 0 | 0 | 0 | 0 | 1 | 0 | 1 | 0 | 0 | 8 |
|  |  |  | 2 | 0 | 0 | 2 | 0 | 0 | 0 | 0 | 0 | 0 | 1 | 0 | 1 | 0 | 0 |  |

*****: hyperintense on STIR sequences

*^†^* STIR sequences not available

**LGMD1D**

| **Patient ID** | **Sex (M=1;F=2)** | **Age (years)** | **Side (right=1;left=2)** | **Sternocleidomastoid** | **Neck extensors** | **Thoracic paraspinal** | **Trapezius** | **Levator scapulae** | **Rhomboids** | **Serratus anterior** | **Supraspinatus** | **Infraspinatus** | **Subscapularis** | **Pectoralis major** | **Pectoralis minor** | **Latissimus dorsi** | **Teres major** | **T1-MRI score** |
| --- | --- | --- | --- | --- | --- | --- | --- | --- | --- | --- | --- | --- | --- | --- | --- | --- | --- | --- |
| 1 | 1 | 42 | 1 | 0 | 0 | 0 | 0 | 0 | 0 | 1 | 0 | 0 | 1 | 0 | 0 | 0 | 0 | 4 |
|  |  |  | 2 | 0 | 0 | 0 | 0 | 0 | 0 | 1 | 0 | 0 | 1 | 0 | 0 | 0 | 0 |  |
| 2 | 2 | 45 | 1 | 0 | 0 | 0 | 0 | 0 | 0 | 1 | 0 | 0 | 0 | 0 | 0 | 0 | 0 | 2 |
|  |  |  | 2 | 0 | 0 | 0 | 0 | 0 | 0 | 1 | 0 | 0 | 0 | 0 | 0 | 0 | 0 |  |
| 3 | 2 | 52 | 1 | 0 | 0 | 0 | 0 | 0 | 0 | 1 | 0 | 0 | 0 | 0 | 0 | 0 | 0 | 2 |
|  |  |  | 2 | 0 | 0 | 0 | 0 | 0 | 0 | 1 | 0 | 0 | 0 | 0 | 0 | 0 | 0 |  |
| 4 | 2 | 70 | 1 | 0 | 0 | 0 | 0 | 0 | 0 | 1 | 1 | 1 | 0 | 0 | 0 | 0 | 0 | 4 |
|  |  |  | 2 | 0 | 0 | 0 | 0 | 0 | 0 | 1 | 0 | 0 | 0 | 0 | 0 | 0 | 0 |  |

**DMD carriers**

| **Patient ID** | **Sex (M=1;F=2)** | **Age (years)** | **Side (right=1;left=2)** | **Sternocleidomastoid** | **Neck extensors** | **Thoracic paraspinal** | **Trapezius** | **Levator scapulae** | **Rhomboids** | **Serratus anterior** | **Supraspinatus** | **Infraspinatus** | **Subscapularis** | **Pectoralis major** | **Pectoralis minor** | **Latissimus dorsi** | **Teres major** | **T1-MRI score** |
| --- | --- | --- | --- | --- | --- | --- | --- | --- | --- | --- | --- | --- | --- | --- | --- | --- | --- | --- |
| 1 | 2 | 37 | 1 | 0 | 0 | 0 | 2 | 0 | 1 | 2 | 3 | 3 | 3 | 2 | 3 | 3 | 3 | 53 |
|  |  |  | 2 | 1 | 0 | 0 | 3 | 0 | 2 | 2 | 3 | 3 | 3 | 2 | 3 | 3 | 3 |  |
| 2 | 2 | 46 | 1 | 0 | 0 | 1 | 2 | 1 | 0 | 2 | 1 | 2 | 2 | 3 | 3 | 2 | 2 | 44 |
|  |  |  | 2 | 0 | 0 | 1 | 2 | 1 | 3 | 3 | 1 | 1 | 1 | 3 | 3 | 3 | 1 |  |
| 3 | 2 | 54 | 1 | 0 | 0 | 0 | 0 | 0 | 0 | 0 | 0 | 0 | 0 | 0 | 0 | 0 | 0 | 1 |
|  |  |  | 2 | 0 | 0 | 0 | 0 | 0 | 0 | 0 | 0 | 0 | 1 | 0 | 0 | 0 | 0 |  |

**Hereditary Myopathy with Early Respiratory Failure**

| **Patient ID** | **Sex (M=1;F=2)** | **Age (years)** | **Side (right=1;left=2)** | **Sternocleidomastoid** | **Neck extensors** | **Thoracic paraspinal** | **Trapezius** | **Levator scapulae** | **Rhomboids** | **Serratus anterior** | **Supraspinatus** | **Infraspinatus** | **Subscapularis** | **Pectoralis major** | **Pectoralis minor** | **Latissimus dorsi** | **Teres major** | **T1-MRI score** |
| --- | --- | --- | --- | --- | --- | --- | --- | --- | --- | --- | --- | --- | --- | --- | --- | --- | --- | --- |
| 1 | 1 | 32 | 1 | 2 | 2 | 2 | 0 | 0 | 2 | 2 | 0 | 0 | 1 | 0 | 3 | 0 | 0 | 27 |
|  |  |  | 2 | 2 | 2 | 2 | 0 | 0 | 2 | 2 | 0 | 0 | 1 | 0 | 2 | 0 | 0 |  |
| 2 | 2 | 57 | 1 | 1 | 0 | 1 | 0 | 0 | 1 | 2 | 0 | 0 | 0 | 1 | 1 | 1 | 0 | 16 |
|  |  |  | 2 | 0 | 1 | 1 | 0 | 0 | 1 | 2 | 0 | 0 | 0 | 1 | 1 | 1 | 0 |  |
| 3 | 2 | 54 | 1 | 3 | 1 | 1 | 0 | 3 | 2 | 3 | 1 | 2 | 1 | 2 | 3 | 0 | 0 | 43 |
|  |  |  | 2 | 3 | 1 | 1 | 0 | 3 | 2 | 3 | 2 | 2 | 1 | 2 | 1 | 0 | 0 |  |

**Sporadic Late-Onset Nemaline Myopathy**

| **Patient ID** | **Sex (M=1;F=2)** | **Age (years)** | **Side (right=1;left=2)** | **Sternocleidomastoid** | **Neck extensors** | **Thoracic paraspinal** | **Trapezius** | **Levator scapulae** | **Rhomboids** | **Serratus anterior** | **Supraspinatus** | **Infraspinatus** | **Subscapularis** | **Pectoralis major** | **Pectoralis minor** | **Latissimus dorsi** | **Teres major** | **T1-MRI score** |
| --- | --- | --- | --- | --- | --- | --- | --- | --- | --- | --- | --- | --- | --- | --- | --- | --- | --- | --- |
| 1 | 1 | 64 | 1 | 0 | 3 | 3 | 2 | 3 | 2 | 3 | 0 | 0 | **3*** | 1 | 0 | 1 | 1 | 38 |
|  |  |  | 2 | 0 | 3 | 3 | 2 | 3 | 1 | 3 | 0 | 0 | **3*** | 1 | 0 | 1 | 2 |  |
| 2 | 1 | 58 | 1 | 2 | 2 | 2 | **2*** | 2 | 3 | 3 | **1*** | **1*** | 3 | **2*** | **3*** | 3 | 3 | 46 |
|  |  |  | 2 | 2 | 2 | 2 | **2*** | 2 | 3 | 3 | **2*** | **1*** | 3 | **3*** | **3*** | 3 | 3 |  |

*****: hyperintense on STIR sequences

**MYH7-related myopathy**

| **Patient ID** | **Sex (M=1;F=2)** | **Age (years)** | **Side (right=1;left=2)** | **Sternocleidomastoid** | **Neck extensors** | **Thoracic paraspinal** | **Trapezius** | **Levator scapulae** | **Rhomboids** | **Serratus anterior** | **Supraspinatus** | **Infraspinatus** | **Subscapularis** | **Pectoralis major** | **Pectoralis minor** | **Latissimus dorsi** | **Teres major** | **T1-MRI score** |
| --- | --- | --- | --- | --- | --- | --- | --- | --- | --- | --- | --- | --- | --- | --- | --- | --- | --- | --- |
| 1 | 2 | 67 | 1 | 3 | 1 | 1 | 2 | 2 | 1 | 3 | 1 | 1 | 1 | 2 | 2 | 3 | 1 | 46 |
|  |  |  | 2 | 3 | 1 | 1 | 2 | 1 | 1 | 3 | 1 | 1 | 1 | 2 | 2 | 2 | 1 |  |
| 2 | 1 | 46 | 1 | 0 | 0 | 0 | 2 | 0 | 0 | 1 | 0 | 0 | 1 | 0 | 0 | 0 | 0 | 7 |
|  |  |  | 2 | 0 | 0 | 0 | 2 | 0 | 0 | 1 | 0 | 0 | 0 | 0 | 0 | 0 | 0 |  |

**TPM2-related myopathy**

| **Patient ID** | **Sex (M=1;F=2)** | **Age (years)** | **Side (right=1;left=2)** | **Sternocleidomastoid** | **Neck extensors** | **Thoracic paraspinal** | **Trapezius** | **Levator scapulae** | **Rhomboids** | **Serratus anterior** | **Supraspinatus** | **Infraspinatus** | **Subscapularis** | **Pectoralis major** | **Pectoralis minor** | **Latissimus dorsi** | **Teres major** | **T1-MRI score** |
| --- | --- | --- | --- | --- | --- | --- | --- | --- | --- | --- | --- | --- | --- | --- | --- | --- | --- | --- |
| 1 | 1 | 46 | 1 | 0 | 2 | 1 | 0 | 0 | 1 | 1 | 0 | 0 | 0 | 0 | 0 | 1 | 0 | 12 |
|  |  |  | 2 | 0 | 2 | 1 | 0 | 0 | 1 | 1 | 0 | 0 | 0 | 0 | 0 | 1 | 0 |  |
| 2 | 1 | 13 | 1 | 0 | 1 | 2 | 0 | 0 | 0 | 0 | 0 | 0 | 0 | 1 | 0 | 1 | 0 | 10 |
|  |  |  | 2 | 0 | 1 | 2 | 0 | 0 | 0 | 0 | 0 | 0 | 0 | 1 | 0 | 1 | 0 |  |

**OTHER**

|  | **Patient ID** | **Sex (M=1;F=2)** | **Age (years)** | **Side (right=1;left=2)** | **Sternocleidomastoid** | **Neck extensors** | **Thoracic paraspinal** | **Trapezius** | **Levator scapulae** | **Rhomboids** | **Serratus anterior** | **Supraspinatus** | **Infraspinatus** | **Subscapularis** | **Pectoralis major** | **Pectoralis minor** | **Latissimus dorsi** | **Teres major** | **T1-MRI score** |
| --- | --- | --- | --- | --- | --- | --- | --- | --- | --- | --- | --- | --- | --- | --- | --- | --- | --- | --- | --- |
| Anti-SRP Myopathy*^†^* | 1 | 2 | 54 | 1 | 0 | 0 | 0 | 0 | 0 | 0 | 1 | 0 | 0 | 0 | 0 | 0 | 0 | 0 | 5 |
|  |  |  |  | 2 | 0 | 0 | 0 | 0 | 0 | 0 | 1 | 0 | 1 | 2 | 0 | 0 | 0 | 0 |  |
| FLNC-Mutated MFM | 2 | 1 | 70 | 1 | 0 | 2 | 3 | 1 | 0 | 1 | 1 | 0 | 0 | 0 | 0 | 0 | 1 | 0 | 18 |
|  |  |  |  | 2 | 0 | 2 | 3 | 1 | 0 | 1 | 1 | 0 | 0 | 0 | 0 | 0 | 1 | 0 |  |
| Nemaline Myopathy | 3 | 2 | 68 | 1 | 2 | 2 | 2 | 1 | 0 | 1 | 2 | 1 | 1 | 2 | 2 | 2 | 2 | 1 | 42 |
|  |  |  |  | 2 | 2 | 2 | 2 | 1 | 0 | 1 | 2 | 1 | 1 | 2 | 2 | 2 | 2 | 1 |  |

*^†^* STIR sequences not available

**Table S2**

Summary of individual muscle scores across all the non-FSHD patients, subdivided by diagnosis. In LGMD2L asymmetric involvement is a feature, but the teres major is the most involved muscle, followed by thoracic paraspinal muscles with relative sparing of trapezius and serratus anterior. In LGMD1D, the serratus anterior is always mildly involved, sometimes together with spinati or subscapularis, and the trapezius is always spared. In manifesting female carriers of dystrophinopathy, asymmetrical involvement is present together with trapezius and serratus anterior involvement, but heavy and early involvement of supra- and infraspinatus, pectoralis minor and subscapularis help in the differential diagnosis with FSHD. In HMERF, the sternocleidomastoid is always affected and the trapezius is always spared. In SLONM, serratus anterior and subscapularis are more affected than trapezius. In *MYH7*-related myopathy, the sternocleidomastoid is heavily affected in the more severe patient and subscapularis involvement would make the diagnosis of FSHD unlikely in the milder one. In *TPM2*-related myopathy, the trapezius is always spared and thoracic paraspinal and neck extensors are affected. STIR hyperintensities could be found only in a minority of non-FSHD patients. Differences between LGMD2A, LGMD2B, AMD and FSHD patterns are discussed in the text.
