# Supplementary material for: Upper Girdle Imaging in Facioscapulohumeral Muscular Dystrophy
Source: PLoS One. 2014 Jun 16;9(6):e100292. doi: 10.1371/journal.pone.0100292 (PMC4059711; doi:10.1371/journal.pone.0100292)
Supplement: Table S3 — Summary of statistical analyses. (DOCX) [file pone.0100292.s004.docx]

**Demographic, clinical and genetic features of FSHD patients according to sex**

|  |  | **Males** |  | **Females** |  |  |  | **Total** |  |
| --- | --- | --- | --- | --- | --- | --- | --- | --- | --- |
|  |  | n=52 |  | n=56 |  | *p-value* |  | n=108 |  |
|  |  |  |  |  |  |  |  |  |  |
| *EcoRI fragment (Kb)* | | 26.7 ± 6.5 |  | 23.1 ± 5.9 |  | <0.01^§^ |  | 24.8 ± 6.4 |  |
|  |  |  |  |  |  |  |  |  |  |
| *Age (years)* | | 38.1 ± 13.3 |  | 44.4 ± 15.6 |  | 0.027^§^ |  | 41.4 ± 14.8 |  |
|  |  |  |  |  |  |  |  |  |  |
| *Clinical Severity Score (CSS)* | | 3 (1.5-3.5) |  | 3 (1.5-4.0) |  | 0.495*^†^* |  | 3 (1.5-3.5) |  |
|  |  |  |  |  |  |  |  |  |  |
| *T1-MRI score* | | 17 (7-29) |  | 19 (11-34) |  | 0.618*^†^* |  | 19 (9-30) |  |
|  | |  |  |  |  |  |  |  |  |
| *N. of muscles affected* | | 5.5 ± 3.1 |  | 6.1 ± 2.9 |  | 0.296^§^ |  | 5.8 ± 3.0 |  |

Values are means ± standard deviation or medians (interquartile range 25^th^ and 75^th^ percentiles)

^†^ Wilcoxon-Mann-Whitney test; ^§^ t-test

| **Asymmetry** |  | **Right** | |  | **Left** | |  |  |
| --- | --- | --- | --- | --- | --- | --- | --- | --- |
|  |  |  |  |  |  |  |  |  |
|  |  | mean ± SD | range |  | mean ± SD | range |  | *p-value^†^* |
|  |  |  |  |  |  |  |  |  |
| *T1-MRI score per side* | | 11.13 ± 7.49 | 0-30 |  | 9.89 ± 7.11 | 0-29 |  | <0.001 |
|  |  |  |  |  |  |  |  |  |
| *N. of muscles affected per side* | | 5.49 ± 2.98 | 0-13 |  | 5.23 ± 3.07 | 0-13 |  | <0.01 |
|  |  |  |  |  |  |  |  |  |
| *T1-MRI score per side and per muscle* | | mean ± SD | range |  | mean ± SD | range |  | *p-value^‡^* |
|  |  |  |  |  |  |  |  |  |
|  | *serratus anterior* | 1.59 ± 0.97 | 0-3 |  | 1.30 ± 0.95 | 0-3 |  | <0.0001 |
|  | *pectoralis minor* | 0.91 ± 1.17 | 0-3 |  | 0.72 ± 1.03 | 0-3 |  | <0.01 |
|  | *pectoralis major* | 1.37 ± 1.01 | 0-3 |  | 1.23 ± 1.02 | 0-3 |  | 0.01 |
|  | *trapezius* | 2.41 ± 0.88 | 0-3 |  | 2.21 ± 0.91 | 0-3 |  | 0.054 |
|  | *rhomboids* | 1.04 ± 1.28 | 0-3 |  | 0.83 ± 1.13 | 0-3 |  | 0.071 |
|  | *levator scapulae* | 0.45 ± 1.03 | 0-3 |  | 0.28 ± 0.75 | 0-3 |  | 0.092 |
|  | *latissimus dorsi* | 1.44 ± 1.15 | 0-3 |  | 1.35 ± 1.09 | 0-3 |  | 0.263 |
|  | *infraspinatus* | 0.05 ± 0.25 | 0-2 |  | 0.03 ± 0.16 | 0-1 |  | 0.500 |
|  | *teres major* | 0.56 ± 0.93 | 0-3 |  | 0.63 ± 1.04 | 0-3 |  | 0.832 |
|  | *thoracic paraspinal* | 0.65 ± 0.92 | 0-3 |  | 0.63 ± 0.91 | 0-3 |  | 1.000 |
|  | *subscapularis* | 0.03 ± 0.16 | 0-1 |  | 0.02 ± 0.13 | 0-1 |  | 1.000 |
|  | *supraspinatus* | 0.03 ± 0.16 | 0-1 |  | 0.05 ± 0.32 | 0-3 |  | 1.000 |
|  | *sternocleidomastoid* | 0.41 ± 0.91 | 0-3 |  | 0.42 ± 0.89 | 0-3 |  | 1.000 |
|  | *neck extensors* | 0.19 ± 0.54 | 0-2 |  | 0.19 ± 0.54 | 0-2 |  | 1.000 |

SD = standard deviation; *^†^* Wilcoxon signed rank test; *^‡^* Sign test

**Multiple linear regression**

|  |  | **β***^†^* | |  | **SE***^‡^* | |  | ***p-value*** | |
| --- | --- | --- | --- | --- | --- | --- | --- | --- | --- |
|  |  |  |  |  |  |  |  |  |  |
| *Age* | | 0.04 | |  | 0.01 | |  | <0.0001 | |
|  |  |  |  |  |  |  |  |  |  |
| *Gender* | | -0.43 | |  | 0.33 | |  | 0.192 | |
|  |  |  |  |  |  |  |  |  |  |
| *EcoRI fragment length* | | -0.09 | |  | 0.26 | |  | <0.001 | |

*^†^* Unstandardized regression coefficient; *^‡^* Standard error
