# Supplementary material for: Upper Girdle Imaging in Facioscapulohumeral Muscular Dystrophy
Source: PLoS One. 2014 Jun 16;9(6):e100292. doi: 10.1371/journal.pone.0100292 (PMC4059711; doi:10.1371/journal.pone.0100292)
Supplement: Table S4 — Asymmetric involvement and STIR hyperintensities in FSHD and other myopathies. (DOCX) [file pone.0100292.s005.docx]

|  |  |  |  |  |  |  |  | **FSHD** |  | **Other myopathies** |  | **p-value^†^** |
| --- | --- | --- | --- | --- | --- | --- | --- | --- | --- | --- | --- | --- |
|  |  |  |  |  |  |  |  |  |  |  |  |  |
|  |  |  |  |  |  |  |  |  |  |  |  |  |
| Patients with minor asymmetry of at least one muscle  (side-to-side difference of at least 1 point) | | | | | | |  | 89 % (96/108) |  | 62 % (28/45) |  | <0.001 |
|  |  |  |  |  |  |  |  |  |  |  |  |  |
| Patients with major asymmetry of at least one muscle  (side-to-side difference of at least 2 points) | | | | | | |  | 47 % (51/108) |  | 20 % (9/45) |  | <0.001 |
|  |  |  |  |  |  |  |  |  |  |  |  |  |
| Patients with at least one muscle affected (score > 0)  and sparing of the contralateral | | | | | | |  | 61 % (66/108) |  | 29 % (13/45) |  | <0.001 |
|  |  |  |  |  |  |  |  |  |  |  |  |  |
| Patients with at least one muscle affected with score > 1  and sparing of the contralateral | | | | | | |  | 37% (40/108) |  | 13 % (6/45) |  | <0.001 |
|  |  |  |  |  |  |  |  |  |  |  |  |  |
|  |  |  |  |  |  |  |  |  |  |  |  |  |
| Patients with at least one muscle affected on STIR sequences | | | | | | |  | 31 % (34/108) |  | 16 % (7/43) |  | <0.001 |
|  |  |  |  |  |  |  |  |  |  |  |  |  |
| Overall number of muscles affected on STIR sequences | | | | | | |  | 2% (60/3024) |  | 1,5 % (18*/1204) |  | <0.001 |
|  |  |  |  |  |  |  |  |  |  |  |  |  |
| Overall number of muscles affected on STIR sequences  and spared on T1-W sequences | | | | | | |  | 0,26 % (8/3024) |  | 0,08 % (1/1204) |  | <0.001 |
|  |  |  |  |  |  |  |  |  |  |  |  |  |
|  |  |  |  |  |  |  |  |  |  |  |  |  |
| * 10/18 belong to the same patient affected by SLONM | | | |  |  |  |  |  |  |  |  |  |
| ^†^ Fisher’s exact test | |  |  |  |  |  |  |  |  |  |  |  |

**Table S4**. Asymmetric involvement and STIR hyperintensities in FSHD and other myopathies
